# Research trends and topics on sepsis immunosuppression: a bibliometric and visual analysis of global research from 2004 to 2024

**DOI:** 10.3389/fmed.2025.1615753

**Published:** 2025-08-04

**Authors:** Ji Yin, Yantong Wan, Guangqin Chen, Nanhong Li, Fei Liu, Haihua Luo, Yijing Li, Shan Li, Yong Jiang

**Affiliations:** ^1^Guangdong Provincial Key Laboratory of Proteomics, School of Basic Medical Sciences, Southern Medical University, Guangzhou, China; ^2^Institute of Infection and Immunity, Henan Academy of Innovations in Medical Science, Zhengzhou, China; ^3^Henan Key Laboratory of Critical Care Medicine, Department of Emergency Medicine, The First Affiliated Hospital, Zhengzhou University, Zhengzhou, China; ^4^Henan International Joint Laboratory of Infection and Immunity, The First Affiliated Hospital, Zhengzhou University, Zhengzhou, China; ^5^Department of Respiratory and Critical Care Medicine, The Tenth Affiliated Hospital (Dongguan People's Hospital), Southern Medical University, Dongguan, China; ^6^Department of Anesthesiology, The Third Affiliated Hospital of Southern Medical University, Guangzhou, China

**Keywords:** sepsis, immunosuppression, bibliometric analysis, immunoparalysis, clinical treatment

## Abstract

**Background:**

Sepsis, a condition characterized by critical organ impairment resulting from dysregulated response to infection, remains a leading cause of mortality globally. Immunosuppression significantly dictates the clinical trajectory and ultimate prognosis in sepsis. Research on sepsis-related immunosuppression has gained substantial attention in recent years. However, a comprehensive and objective analysis of the current state of research remains scarce. This study employs bibliometric techniques and knowledge mapping to provide a visual analysis of trends and developments in sepsis immunosuppression, offering a systematic overview for researchers in the field.

**Objectives:**

This study aims to: (1) Quantitatively characterize global publication trends, key contributors (countries/institutions/journals/authors), and collaborative networks in sepsis immunosuppression research (2004–2024); (2) Identify current hotspots and future directions in this field.

**Materials and methods:**

Articles and reviews related to sepsis immunosuppression were sourced from the Web of Science Core Collection *via* subject-specific searches. Bibliometric analyses were executed using Excel 365, CiteSpace, VOSviewer, and Bibliometrix (R-Studio’s R-Tool).

**Results:**

From January 1, 2004, to March 22, 2024, 3,082 articles on sepsis immunosuppression authored by 16,545 individuals across 116 institutions from 30 countries were identified. Collaborative network analysis highlights the United States as the leading contributor, with key institutions including the University of Florida and Washington University. Strong international collaboration is evident, particularly between the United States and China, as well as the United States and Germany. Frontiers in Immunology emerged as the most prolific journal, while Journal of Immunology garnered the highest co-citation frequency. Guillaume Monneret has published the greatest number of relevant articles, while Richard S. Hotchkiss is the most frequently co-cited author. The research predominantly focuses on immunology, molecular biology, and clinical treatments. Keyword analysis suggests that immune cells, cell death, and personalized clinical treatments are emerging research hotspots in the field.

**Conclusion:**

This bibliometric analysis maps evolving trends and forecasts emerging hotspots in sepsis immunosuppression research, providing direction for future studies.

## 1 Introduction

Sepsis, characterized by life-threatening organ dysfunction resulting from a dysregulated host response to infection, remains a significant global public health challenge and is the leading cause of mortality among intensive care unit (ICU) patients (1–3). It can result from virtually any infecting organism, with a patient’s susceptibility to infection being the primary factor contributing to sepsis development (4). Despite improvements in early detection, prevention, and treatment, the incidence and mortality rates of sepsis remain alarmingly high (5, 6).

The pathogenesis of sepsis is complex and variable. Although its precise mechanisms remain unclear, the immune response plays a pivotal role in the progression and outcomes of sepsis, involving both pro-inflammatory and anti-inflammatory components, which ultimately disrupt homeostasis (7). Initially, sepsis is marked by a hyperinflammatory response, but a significant number of patients progress to a prolonged state of immunosuppression, which is closely linked to increased late-stage mortality (8, 9). Despite decades of research and clinical efforts, effective therapeutic interventions targeting sepsis-induced immunosuppression have yet to be identified, highlighting the urgent need for innovative treatment strategies (10). The challenges stem from the complexity of the immune response during sepsis and the difficulty in developing therapies that can restore immune balance.

As global scientific output continues to grow, bibliometric analysis has become an essential tool for researchers to understand research dynamics. Bibliometrics applies quantitative and computational linguistics techniques to assess the evolution of disciplines, encompassing three key phases: multi-source data curation from authoritative databases, multi-dimensional analytical processing (including publication trends, authorship and collaboration, geographical and institutional analysis, journal analysis, citation analysis, and keyword analysis), and knowledge mapping *via* advanced visualization tools (11, 12). By utilizing the Web of Science Core Collection (WoSCC), this study aims to provide a comprehensive overview of the current state of sepsis immunosuppression research, exploring trends and forecasting potential research hotspots. Ultimately, it seeks to offer insights that may guide future research directions and therapeutic innovations in the management of sepsis immunosuppression.

## 2 Materials and methods

### 2.1 Data sources and search strategies

The WoSCC is a standardized, comprehensive database for literature searches. Thus, an extensive search was conducted from 2004 to 2024 within the WoSCC, focusing on original articles and reviews.

All searches were performed on March 22, 2024, to mitigate any bias from recent database updates. In WoSCC, “TS” denotes “Topic Sentence,” and the search strategy was constructed as follows: TS = (“Sepsis” OR “Septic Shock” OR “Bloodstream Infection” OR “Bloodstream Infections” OR “Infection, Bloodstream” OR “Pyemia” OR “Pyemias” OR “Pyohemia” OR “Pyohemias” OR “Pyaemia” OR “Pyaemias” OR “Septicemia” OR “Septicemias” OR “Blood Poisoning” OR “Blood Poisonings”) AND TS = (“Immune Suppression” OR “Immunosuppression” OR “Immune Tolerance” OR “Immunologic Paralysis” OR “Immune Paralysis” OR “Paralysis by Immunization” OR “Paralysis by Immune System”).

### 2.2 Data collection and analysis

Our inclusion criteria were strictly limited to English-language journal articles and reviews. Exclusion criteria encompassed non-English publications, studies outside the specified timeframe, and other document types (e.g., conference papers, books, and letters). Duplicate records were automatically identified and removed using CiteSpace. Dual independent screening was performed by two reviewers (Y W, J Y) to ensure retained literature directly addressed sepsis immunosuppression.

**Figure 1:**
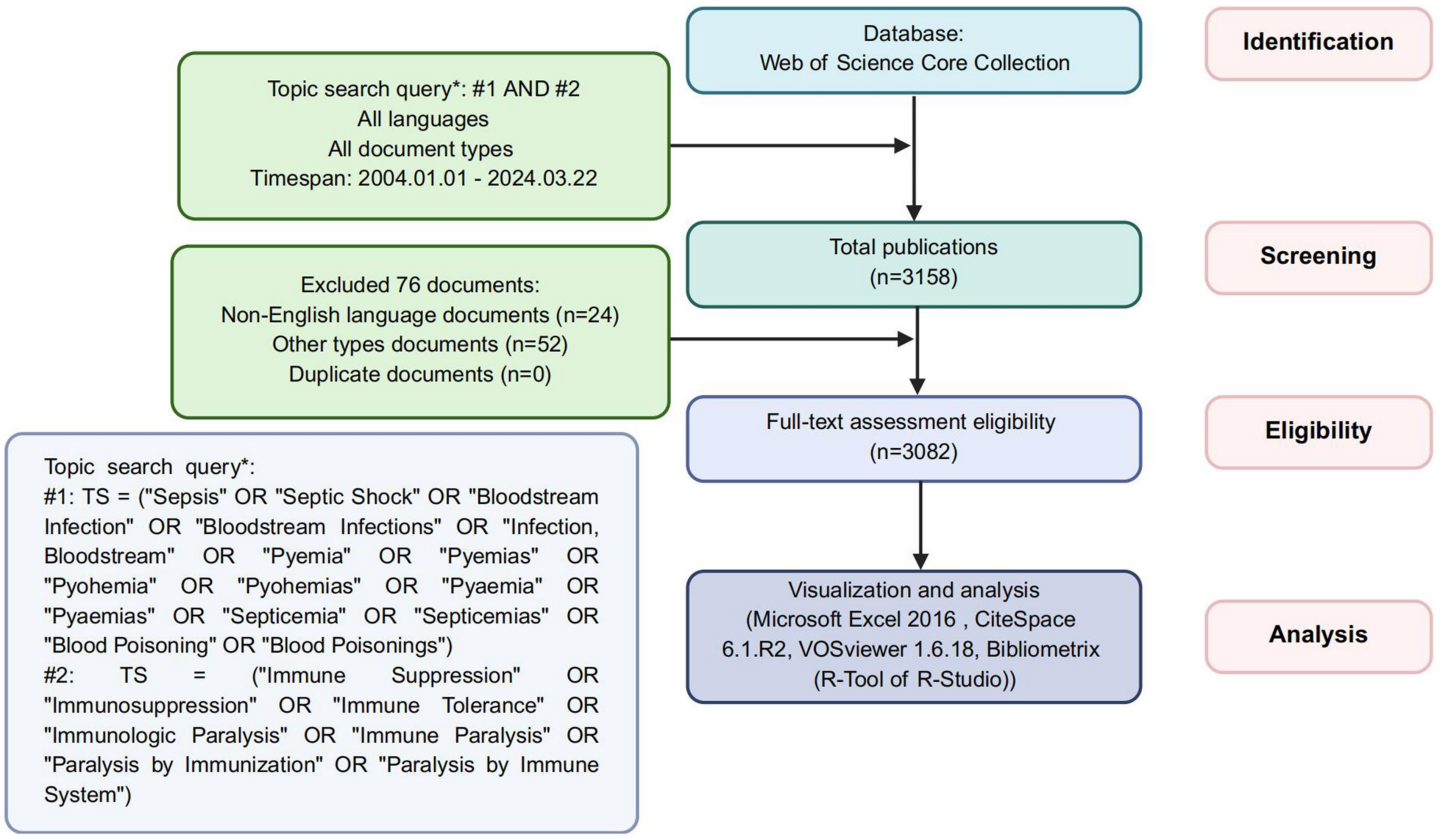
The flowchart of screening process.

The screening process (Figure 1) began with 3,158 identified records. After excluding 76 irrelevant publications, 3,082 articles were retained for analysis. These publications were exported as plain text files containing complete records and cited references. The extracted metadata encompassed titles, keywords, publication dates, geographic origins, authorship, institutional affiliations, source journals, cited references, and citation metrics. Data analysis was performed using Microsoft Excel 2016, CiteSpace 6.1.R2, VOSviewer 1.6.18, and Bibliometrix (R package in R-Studio).

To visualize and analyze the data, tasks were distributed across specialized platforms. Microsoft Excel 2016 was used to track publication and citation trends over time. CiteSpace, a Java-based tool grounded in set-theoretic principles (13, 14), enabled normalization and similarity quantification of knowledge units, facilitating advanced visualizations of geographic distributions, institutional clustering, journal networks, authorship patterns, reference clusters, and keyword burst detection. VOSviewer was employed for additional analyses, creating bibliometric maps that prioritized visual clarity and structural elegance (15). This tool specifically examined collaborative networks across nations, institutions, journals, and authors, alongside keyword co-occurrence patterns. Finally, Bibliometrix provided further visual analysis of country distributions, journal impacts, and keyword dynamics through R-based computational frameworks.

## 3 Results

### 3.1 Evolution in annual research productivity

**Figure 2:**
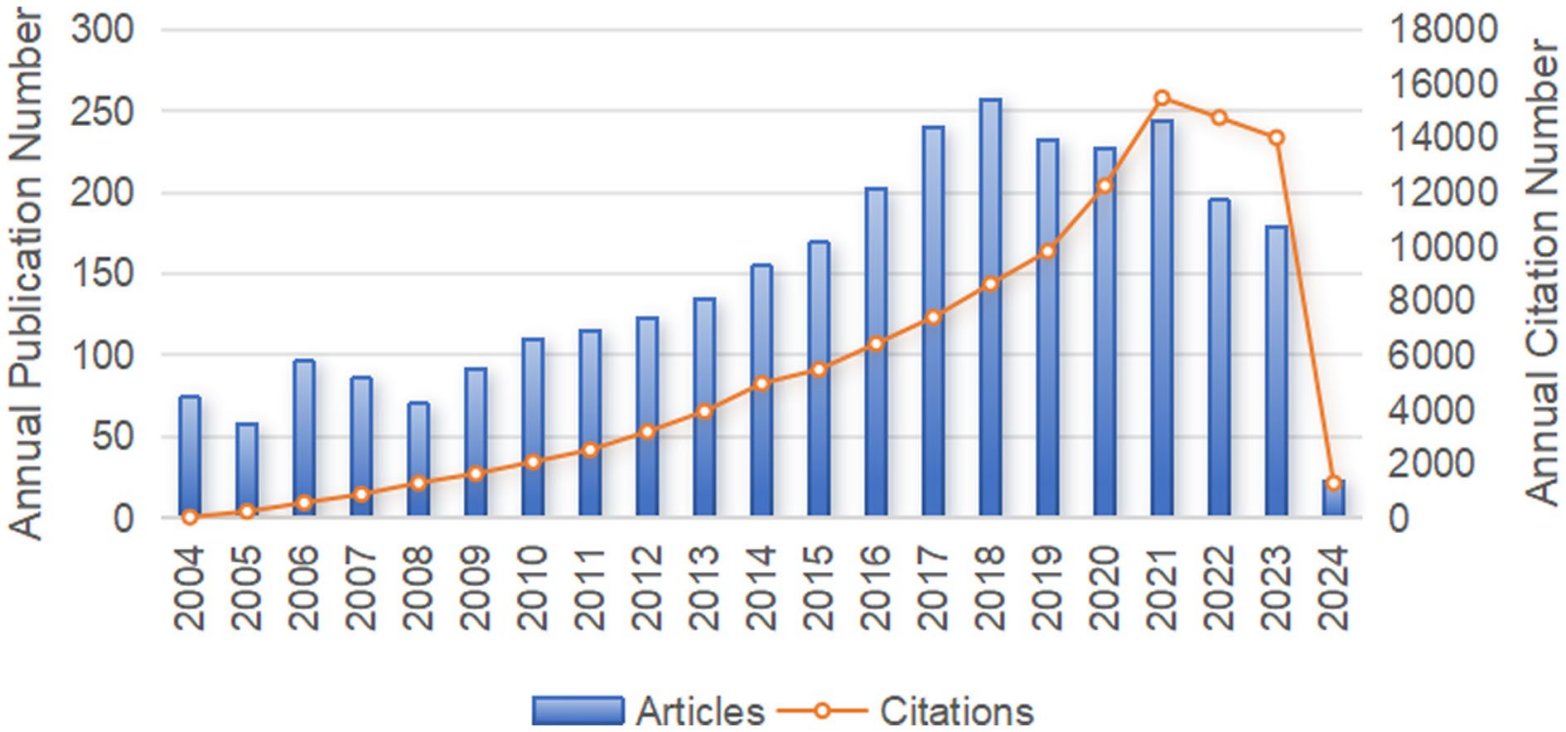
The annual number of publication volume and citation frequency in sepsis immunosuppression from 2004 to 2024.

Our systematic search procured 3,082 pertinent publications on sepsis immunosuppression from the WoSCC database between 2004 and 2024. As depicted in Figure 2, the annual publication volume exhibited a steady upward trend. From 2004 to 2009, the number of articles published annually remained below 100. However, from 2010 on, the annual publication volume consistently exceeded 100, with the highest output of 257 publications in 2018. The annual citation frequency of sepsis immunosuppression research followed a similar upward trajectory, reaching a peak of 15,477 citations in 2021. These trends underscore the growing interest in sepsis and immunosuppression, with an increasing number of prospective studies emerging in this field.

### 3.2 Analysis of countries

**Figure 3:**
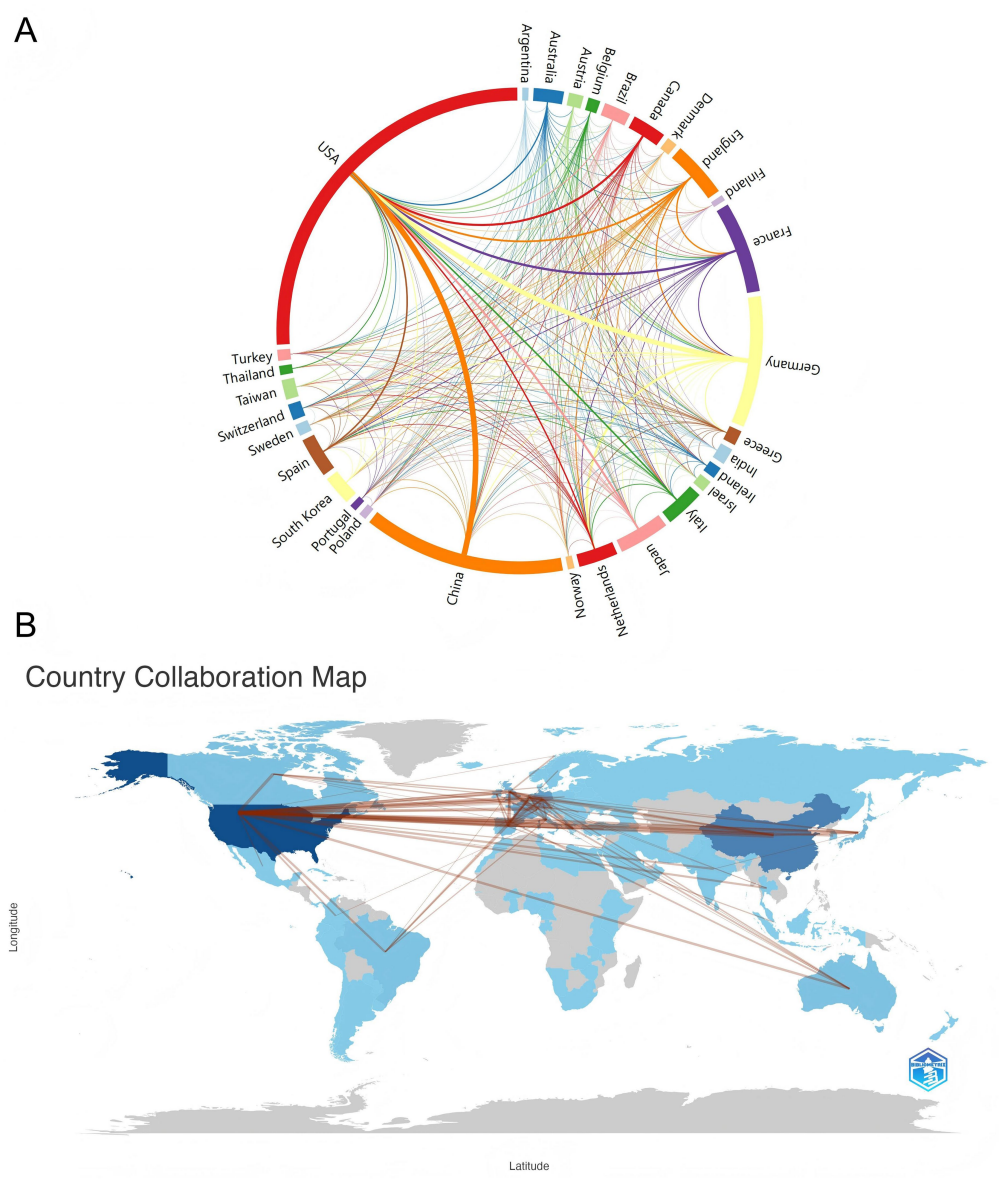
Visualizing cooperation between countries/regions. **(A)** The network map of cooperation between countries. Lines between nodes represent cooperation, and the color of the lines indicates the first cooperation year. The thickness between the connecting lines represents the closeness of the ties. **(B)** Country collaboration map. The links between countries represent their collaborations.

Research on sepsis immunosuppression involved contributions from 30 countries. The top 10 countries were ranked based on the number of publications and citations (Table 1). The United States led the field with 1,052 publications, the only country surpassing 1,000 papers, followed by China with 550 publications. All other countries published fewer than 500 articles. In terms of citations and total link strength, the United States and Germany ranked first and second, respectively. Notably, while the United States published twice as many articles as China, it garnered 55,252 citations—five times more than China’s 10,101. This discrepancy suggests a divergence between the quantity and quality of publications in China. Further analysis of international collaboration revealed distinct patterns, which are visually represented in the chord diagram (Figure 3A). The circle’s circumference corresponds to the volume of publications from each country, while the thickness of the connecting lines indicates the level of collaboration. The United States, China, and Germany were the most prolific contributors, with the strongest collaboration occurring between China and the United States, followed by Germany and the United States. Figure 3B illustrates the global collaboration network, clearly showing that North America maintains close ties with both Europe and East Asia, highlighting the central role of the United States in the global research network.

**Table 1:** Top 10 countries in terms of the number of publications and citations.

Rank	Country	Documents	Country	Citations	Country	Total link strength
1	USA	1,052	USA	55,252	USA	430
2	China	550	Germany	16,358	Germany	240
3	Germany	357	France	15,336	England	165
4	France	248	China	10,101	Netherlands	155
5	England	154	England	8,300	France	149
6	Japan	138	Netherlands	8,153	Spain	128
7	Spain	109	Canada	7,473	China	124
8	Italy	107	Spain	5,295	Italy	118
9	Netherlands	107	Australia	4,111	Canada	92
10	Canada	93	Japan	3,620	Japan	79

### 3.3 Analysis of institutions

**Figure 4:**
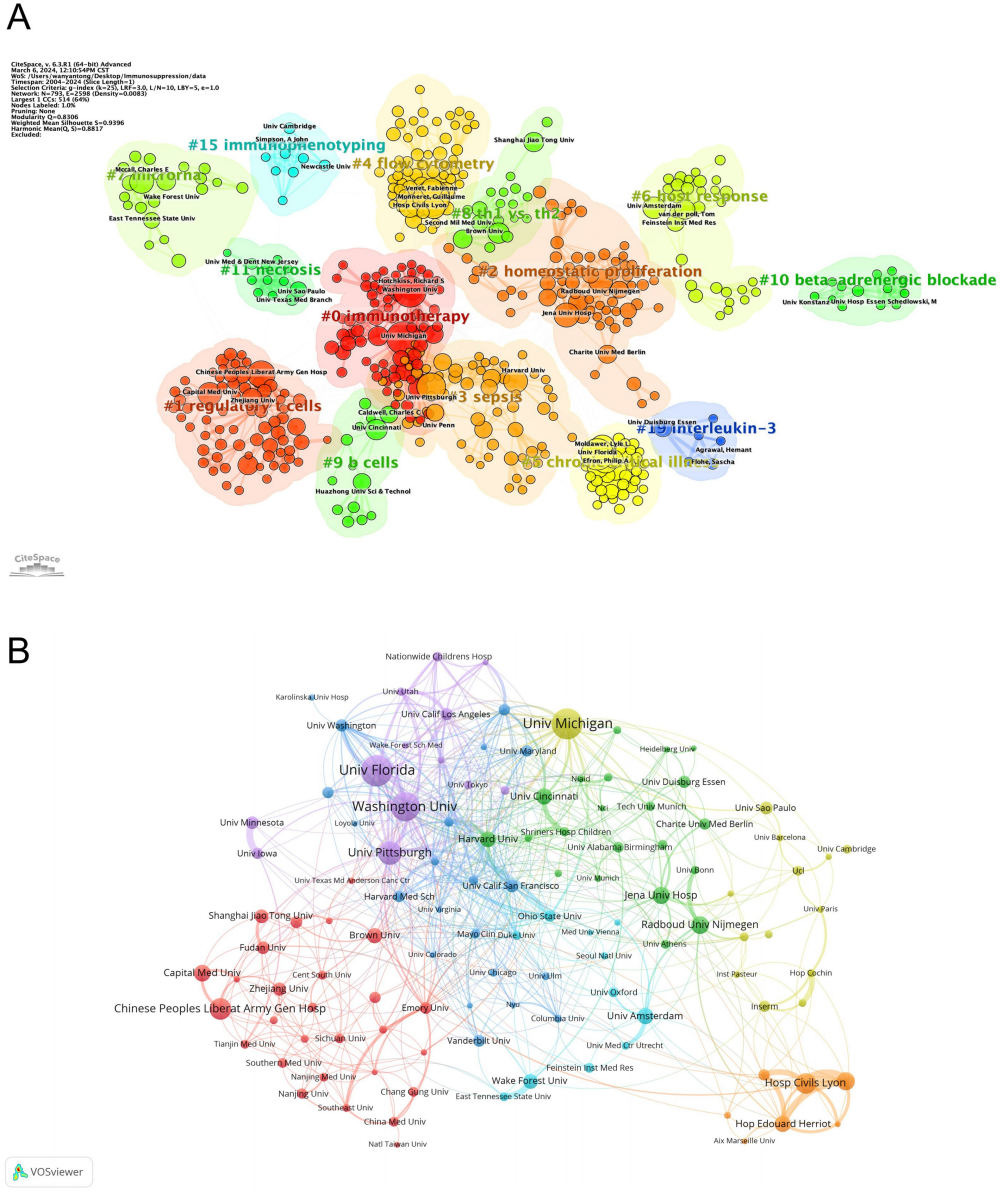
Analysis of institutions related to sepsis-induced immunosuppression. **(A)** Clustering of institutions based on similarity of research topics. **(B)** Collaborative network visualization of institutions.

Table 2 details the top 10 institutions ranked by publication count and citation frequency. The University of Florida led with 71 publications, followed closely by the University of Michigan with 69. Four of the top 10 institutions ranked by publications were based in the United States. In terms of citation frequency, the University of Washington ranked first with 11,374 citations, followed by the University of Florida (6,896) and Harvard University (5,176). Notably, eight of the ten institutions were from the United States, underscoring the country’s dominant role in sepsis-immunosuppression research. Furthermore, institutions were categorized into distinct clusters in CiteSpace, with keyword labels. Figure 4A reveals 14 clusters related to sepsis immunosuppression, with the largest four being #2 homeostatic proliferation, #0 immunotherapy, #3 sepsis, and #1 regulatory T cells, indicating the frequent usage of these terms among the institutions. To gain deeper insight into the global distribution of sepsis-induced immunosuppression research and identify opportunities for collaboration, institutional cooperation was analyzed using VOSviewer. Figure 4B illustrates seven distinct clusters, each represented by a key institution: the University of Florida and Washington University, the University of Michigan, Jena University Hospital and Radboud University Nijmegen, the University of California San Francisco, the Hospital of Civils Lyon, the University of Amsterdam and Wake Forest University, and the Chinese People’s Liberation Army General Hospital. Notably, all clusters, except for the one represented by the Hospital of Civils Lyon, demonstrated close collaborative ties.

**Table 2:** Top 10 institutions in terms of the number of publications and citations.

Rank	Institution	Documents	Country	Institution	Citations	Country
1	Univ Florida	71	USA	Washington Univ	11,374	USA
2	Univ Michigan	69	USA	Univ Florida	6,896	USA
3	Washington Univ	64	USA	Harvard Univ	5,176	USA
4	Univ Pittsburgh	51	USA	Univ Michigan	4,865	USA
5	Chinese Peoples Liberat Army Gen Hosp	45	China	Univ Pittsburgh	4,696	USA
6	Hosp Civils Lyon	43	France	Univ Washington	4,136	USA
7	Univ Claude Bernard Lyon 1	37	France	Radboud Univ Nijmegen	4,116	Netherlands
8	Radboud Univ Nijmegen	36	Netherlands	Univ Calif San Francisco	3,851	USA
9	Jena Univ Hosp	35	Germany	Hop Edouard Herriot	3,453	France
10	Capital Med Univ	34	China	Univ Texas Med Branch	3,267	USA

### 3.4 Analysis of journals

**Figure 5:**
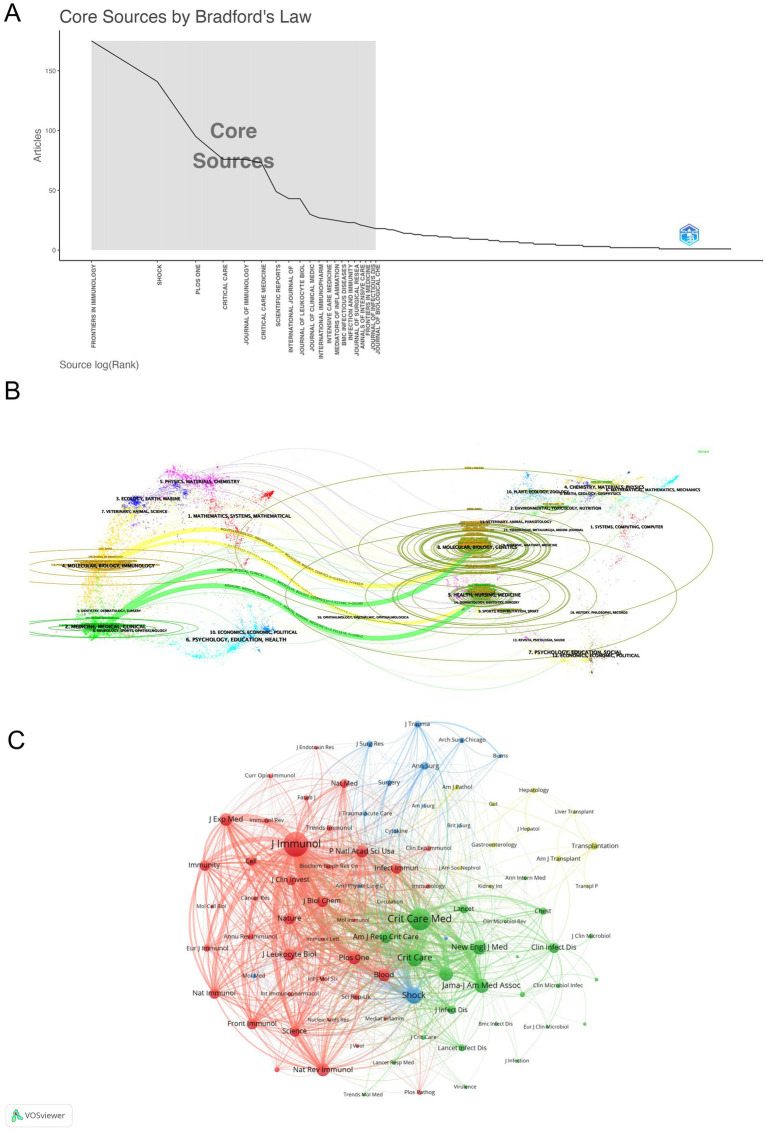
Multidimensional visualization of journals related to sepsis-induced immunosuppression. **(A)** Core-periphery distribution pattern delineated by Bradford’s Law analysis. **(B)** The dual-map overlay of journals, with citing journals (left) and cited journals (right) are connected through colored citation trajectories. **(C)** Visualization of co-journals in VOSviewer.

Journal influence and productivity within the sepsis immunosuppression domain were profiled through visual analyses. Articles related to sepsis immunosuppression were published across 51 academic journals. Table 3 and Figure 5A highlight the journals with the highest publication volumes: Frontiers in Immunology (175 articles), Shock (141), PLOS One (95), Critical Care (76), and Journal of Immunology (76). Regarding co-citations, the top five most frequently cited journals were Journal of Immunology (7,566 citations), Critical Care Medicine (6,214), Critical Care (3,670), Shock (3,665), and Jama-Journal of the American Medical Association (2,815). Notably, two of these journals have accumulated over 5,000 citations, highlighting their substantial influence in the field. Figure 5B presents a dual-map overlay of journals, visually mapping three key dimensions of journal evolution: the spread of individual scholarly journals, the development of citation trajectories, and shifts in research focus. Annotations on the right side of the map indicate source journals generating citations, while labels on the left represent target journals receiving citations. Citation linkages are depicted by colored curves that extend from the citing to the cited journals, illustrating the citation context. Journals that primarily cite belong to fields such as molecular biology, immunology, medicine, and clinical studies, while cited journals are mainly associated with molecular biology, genetics, health, nursing, and medicine. This positioning underscores the significant role these journals play within their respective domains. In addition, a network of co-journals was visualized to explore collaborative relationships among journals publishing literature on sepsis-induced immunosuppression. Figure 5C, generated by VOSviewer, demonstrates the connections between these journals. Four discrete journal clusters were delineated through co-citation network analysis, each representing a specialized research focus: the red cluster, centered on immunology (e.g., Journal of Immunology, Frontiers in Immunology); the green cluster, focused on critical care medicine (e.g., Critical Care Medicine, Critical Care); the blue cluster, associated with surgery (e.g., Shock, Surgery, Annals of Surgery); and the yellow cluster, concentrated on transplantation (e.g., Transplantation, Liver Transplant).

**Table 3:** Top 10 journals in terms of publications and citations.

Rank	Journal	Documents	IF (JCR2023)	JCR quartile	Co-cited journal	Citations	IF (JCR2023)	JCR quartile
1	Frontiers in Immunology	175	5.7	Q1	Journal of Immunology	7,566	3.6	Q2
2	Shock	141	2.7	Q2	Critical Care Medicine	6,214	7.7	Q1
3	PLOS One	95	2.9	Q1	Critical Care	3,670	8.8	Q1
4	Critical Care	76	8.8	Q1	Shock	3,665	2.7	Q2
5	Journal of Immunology	76	3.6	Q2	Jama-Journal of The American Medical Association	2,815	63.5	Q1
6	Critical Care Medicine	73	7.7	Q1	New England Journal of Medicine	2,783	96.3	Q1
7	Scientific Reports	49	3.8	Q1	Intensive Care Medicine	2,660	29.6	Q1
8	International Journal of Molecular Sciences	43	4.9	Q1	Nature Reviews Immunology	2,421	67.7	Q1
9	Journal of Leukocyte Biology	43	3.6	Q2	PLOS One	2,413	2.9	Q1
10	Journal of Clinical Medicine	30	3.0	Q1	Blood	2,374	21.1	Q1

### 3.5 Analysis of authors

**Figure 6:**
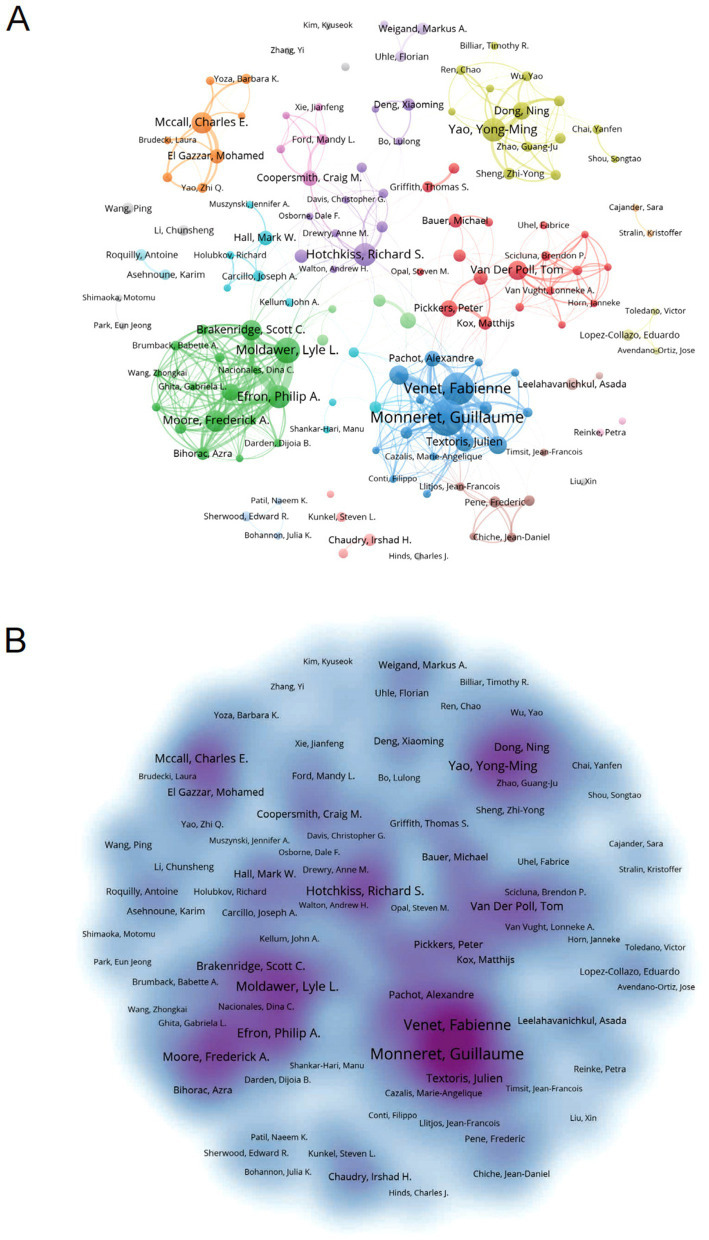
Analysis of authors associated with sepsis-induced immunosuppression. **(A)** Author co-occurrence graph. Nodes of different colors reflect authors in different clusters. Node size indicates co-occurrence frequency and links indicate co-occurrence relationships between authors. **(B)** Density visualization of sepsis-related author links. Stronger correlations are usually presented as dark areas or clusters, while weaker correlations may appear as scattered dots or lighter-colored areas.

Bibliometric analysis identified 16,545 contributors to sepsis immunosuppression research. Table 4 lists the top 10 authors based on publication count and citation frequency in this field. Guillaume Monneret led in terms of publications, with 75 articles, while Richard S. Hotchkiss topped the citation count with 2,660 citations. Figure 6A illustrates the collaborative network among authors, highlighting strong intra-cluster cooperation, exemplified by the influential partnership between Fabienne Venet and Guillaume Monneret, although inter-cluster collaboration remains limited. Figure 6B visualizes co-author relationships, with key centers of collaboration identified, including Fabienne Venet and Guillaume Monneret, Yong-Ming Yao and Ning Dong, and Lyle L Moldawer, Philip A Efron, Scott C Brakenridge, and Frederick A Moore.

**Table 4:** Top 10 most published and 10 most co-cited authors in studies of sepsis immunosuppression.

Rank	Author	Documents	Country	Co-cited author	Citations	Country
1	Monneret, Guillaume	75	France	Hotchkiss, Rs	2,660	USA
2	Venet, Fabienne	64	France	Venet, F	763	France
3	Moldawer, Lyle L.	45	USA	Boomer, Js	645	USA
4	Yao, Yong-Ming	40	China	Singer, M	594	England
5	Efron, Philip A.	38	USA	Monneret, G	561	France
6	Hotchkiss, Richard S.	38	USA	Angus, Dc	506	USA
7	Mccall, Charles E.	34	USA	Bone, Rc	490	USA
8	Moore, Frederick A.	34	USA	Vincent, Jl	457	Belgium
9	Brakenridge, Scott C.	32	USA	Delano, Mj	339	USA
10	Rimmele, Thomas	29	France	Cavaillon, Jm	283	France

**Figure 7:**
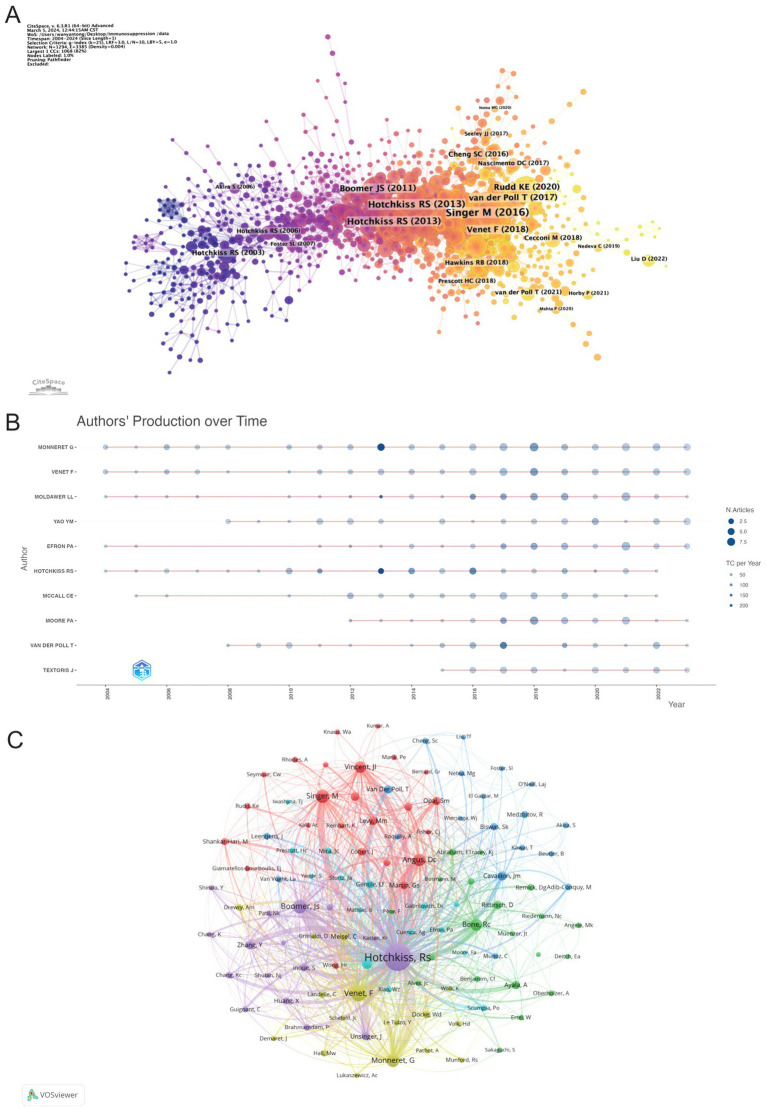
Visualization of authors. **(A)** The graph shows authors active in research on sepsis immunosuppression. The color represents the year of their activity, with yellow indicating the most recent years. **(B)** Visualization of authors’ publications and citations over time. **(C)** The co-authorship network, the node size represents the publications and citation frequencies, and the linkages mean collaborations.

CiteSpace was employed to map authors’ activities over time, as shown in Figure 7A. Authors are color-coded by institution and publication date, with early publications represented in purple, such as those by Hotchkiss RS (2003), while later publications are indicated in yellow. Notably, Hotchkiss RS remained a prominent figure from 2003 to 2013. Figure 7B reveals the number and impact of articles published by authors each year, demonstrating that Monneret G maintained a dominant presence throughout the 2004–2024 period, consistently ranking first in publications. The year 2018 saw a surge in productivity across the field, significantly advancing sepsis immunosuppression research. Furthermore, Figure 7C presents the co-authorship network, highlighting the central roles of Hotchkiss RS, Venet F, and Monneret G in the collaborative landscape of this research.

### 3.6 Analysis of reference

**Figure 8:**
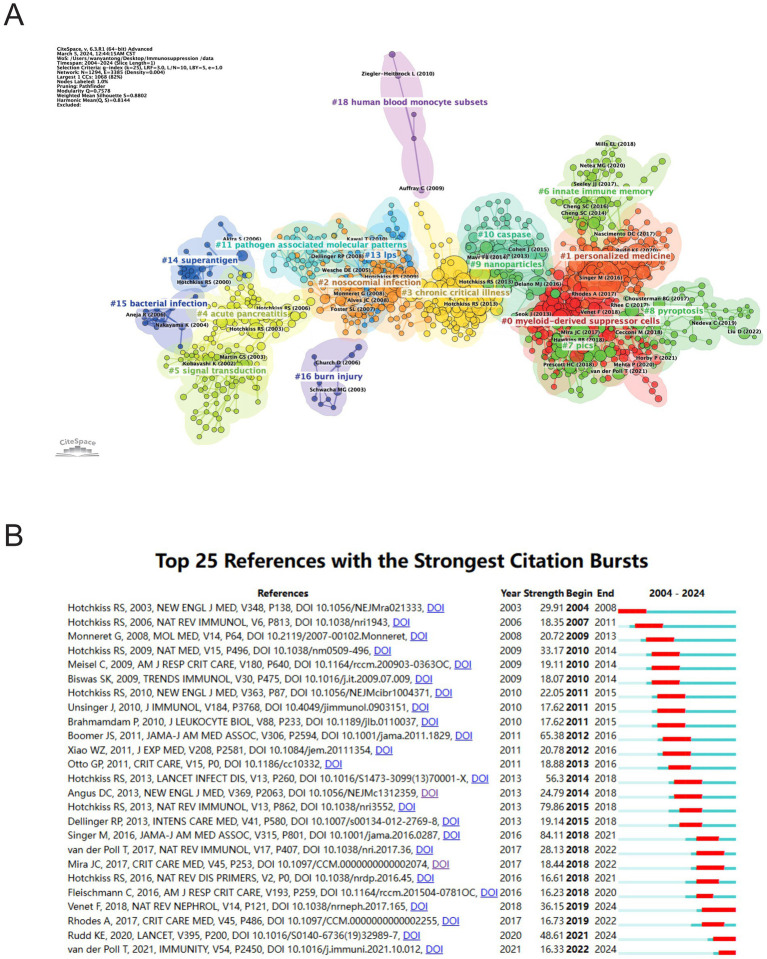
Analysis of references related to sepsis immunosuppression. **(A)** Clustering of references based on the similarity between references. **(B)** The 25 most highly cited documents. The red bar represents the high citation rate for the corresponding year.

Figure 8A illustrates sub-clustering based on similarities among reference articles, resulting in 18 distinct clusters, which highlight shifts in research focus over time. The initial clusters include #14 superantigen, followed by earlier studies on #4 acute pancreatitis, #5 signal transduction, #15 bacterial infection, and #16 burn injury. Emerging clusters, such as #18 human blood monocyte subsets and #11 pathogen-associated molecular patterns, indicate evolving areas of research, with the most recent addition being #8 pyroptosis, reflecting a notable shift in focus. Strong relationships are observed between clusters #7 PICS and #0 myeloid-derived suppressor cells, #2 nosocomial infection and #13 LPS. Meanwhile, #18 human blood monocyte subsets, #16 burn injury, and #6 innate immune memory appear to be developing independently. References with citation surges are those whose citations increase significantly within a given time frame. Table 5 highlights the most frequently cited article, *Genomic responses in mouse models poorly mimic human inflammatory diseases* by Seok J et al., with 2,170 citations (16). Figure 8B presents the top 25 references with the strongest citation bursts. The most significant citation burst (Strength = 84.11) occurred for *The Third International Consensus Definitions for Sepsis and Septic Shock (Sepsis-3)*, authored by Mervyn Singer et al. in 2016, with citation surges from 2018 to 2021 (3). Hotchkiss RS’s 2013 paper, *Sepsis-induced immunosuppression: from cellular dysfunctions to immunotherapy*, published in Nature Reviews Immunology, also experienced a substantial citation burst (Strength = 79.86) (7). Notably, 2018 marked the highest citation burst rate, followed by 2011, suggesting that high-impact papers published in these years played a key role in driving the subsequent research boom. Furthermore, three references—*Advances in the understanding and treatment of sepsis-induced immunosuppression*, *Global, regional, and national sepsis incidence and mortality, 1990–2017: analysis for the Global Burden of Disease Study*, and *The immunology of sepsis*—continue to experience citation bursts into 2024 (17–19).

**Table 5:** Top 10 co-cited references concerning sepsis immunosuppression.

Rank	Title	First author	Journal	Citations	Year
1	Genomic responses in mouse models poorly mimic human inflammatory diseases	Seok, J	Proceedings of the National Academy of Sciences of the United States of America	2,170	2013
2	Sepsis-induced immunosuppression: from cellular dysfunctions to immunotherapy	Hotchkiss, RS	Nature Reviews Immunology	1,570	2013
3	The complex role of estrogens in inflammation	Straub, RH	Endocrine Reviews	1,262	2007
4	Burn wound infections	Church, D	Clinical Microbiology Reviews	1,214	2006
5	Suppression of inflammation by a synthetic histone mimic	Nicodeme, E	Nature	1,205	2010
6	Immunosuppression in Patients Who Die of Sepsis and Multiple Organ Failure	Boomer, JS	Jama-Journal of the American Medical Association	1,173	2011
7	The immunopathology of sepsis and potential therapeutic targets	Van der Poll, T	Nature Reviews Immunology	982	2017
8	Immunosuppression in sepsis: a novel understanding of the disorder and a new therapeutic approach	Hotchkiss, RS	Lancet Infectious Diseases	980	2013
9	Endotoxin tolerance: new mechanisms, molecules, and clinical significance	Biswas, SK	Trends in Immunology	960	2009
10	Ebola hemorrhagic fever	Feldmann, H	Lancet	878	2011

### 3.7 Analysis of hotspots and frontiers

**Figure 9:**
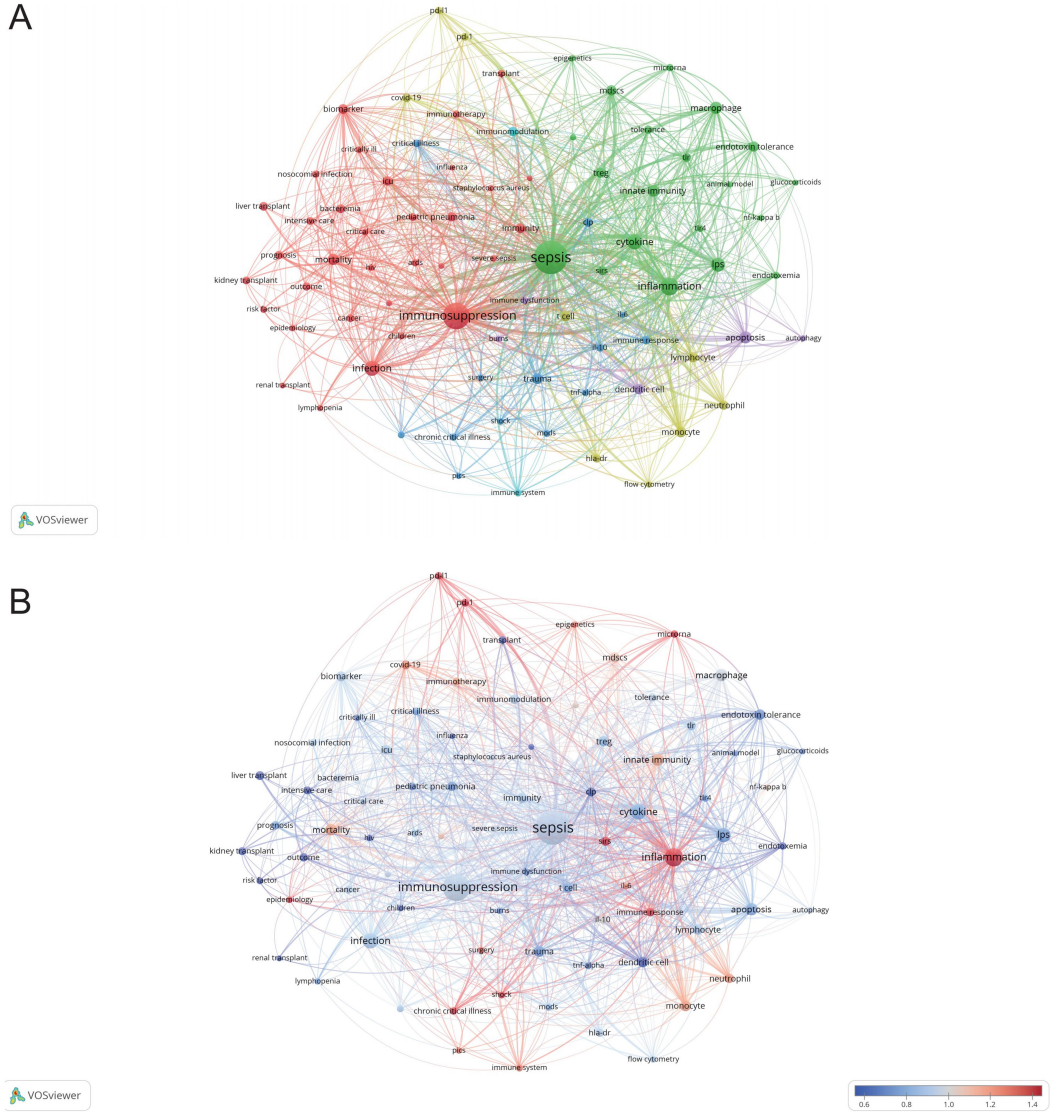
Analysis of keywords related to sepsis immunosuppression. **(A)** The co-occurrence network diagram. The varied colored nodes reflect the keywords in various clusters. The node size means the occurrence frequencies, and the lines mean the co-occurrence relationships between keywords. **(B)** Average normalized citation count. Blue represents the keyword’s smaller average normalized citation count, and red represents the larger average normalized citation count. If two keywords appear in the same line of a corpus file at the same time, it means that the author may have discussed both keywords in the same text, or both keywords appear in the same topic or context. A smaller distance between two keywords usually indicates that the two keywords are more related to each other and may occur in the same sentence, the same paragraph, or in adjacent sentences or paragraphs.

VOSviewer was employed to analyze keywords extracted from 3,082 papers, resulting in the identification of 82 keywords (terms appearing more than 15 times in the titles and abstracts). These keywords were visualized in a co-occurrence network diagram (Figure 9A), where connecting lines indicate co-occurrence relationships between terms. The keywords were categorized into six clusters based on their research focus: the red cluster centered on the clinical diseases and characteristics associated with sepsis immunosuppression (e.g., infection, mortality, liver transplant), the green cluster focused on the pathological mechanisms of sepsis immunosuppression (e.g., inflammation, cytokine, innate immunity), the dark blue cluster related to critical medicine (e.g., shock, PICS, MODS, chronic critical illness), the yellow cluster revolving around immune cells (e.g., monocyte, neutrophil, T cell), the purple cluster emphasizing cell death (e.g., autophagy, apoptosis), and the light blue cluster focusing on the immune system and immunomodulation. The top 20 most frequent keywords are listed in Table 6. Apart from “sepsis” (1,070) and “immunosuppression” (602), other frequently occurring keywords include “inflammation” (230), “cytokine” (150), “infection” (148), and “LPS” (123), underlining their critical roles in sepsis immunosuppression. Figure 9B illustrates the average number of standardized citations for each keyword, with blue representing keywords with lower average citations and red indicating those with higher average citations. As shown in Figure 9B and Table 7, “shock” emerged as the primary topic with the highest number of standardized citations, followed by “chronic critical illness.” Notably, “immune response” ranked third, highlighting the growing importance of immune response research in the field of sepsis immunosuppression.

**Table 6:** Top 20 keywords in terms of frequency concerning sepsis immunosuppression.

Rank	Keyword	Occurrences
1	sepsis	1,070
2	immunosuppression	602
3	inflammation	230
4	cytokine	150
5	infection	148
6	LPS	123
7	macrophage	89
8	apoptosis	89
9	innate immunity	79
10	mortality	71
11	monocyte	67
12	trauma	61
13	MDSCs	59
14	T cell	58
15	endotoxin tolerance	57
16	dendritic cell	57
17	Treg	56
18	biomarker	54
19	immunity	53
20	lymphocyte	51

**Table 7:** Top 20 keywords according to the average number of standardized citations.

Rank	Keyword	Score<Avg. norm. citations>
1	shock	1.6954
2	chronic critical illness	1.6474
3	immune response	1.5741
4	pd-l1	1.538
5	sirs	1.5378
6	pd-1	1.4571
7	microRNA	1.4519
8	epidemiology	1.4164
9	inflammation	1.4128
10	surgery	1.3302
11	pics	1.2904
12	immune system	1.2879
13	epigenetics	1.2869
14	covid-19	1.2515
15	il-6	1.2405
16	neutrophil	1.217
17	immunotherapy	1.2041
18	monocyte	1.1966
19	mortality	1.1811
20	innate immunity	1.1605

**Figure 10:**
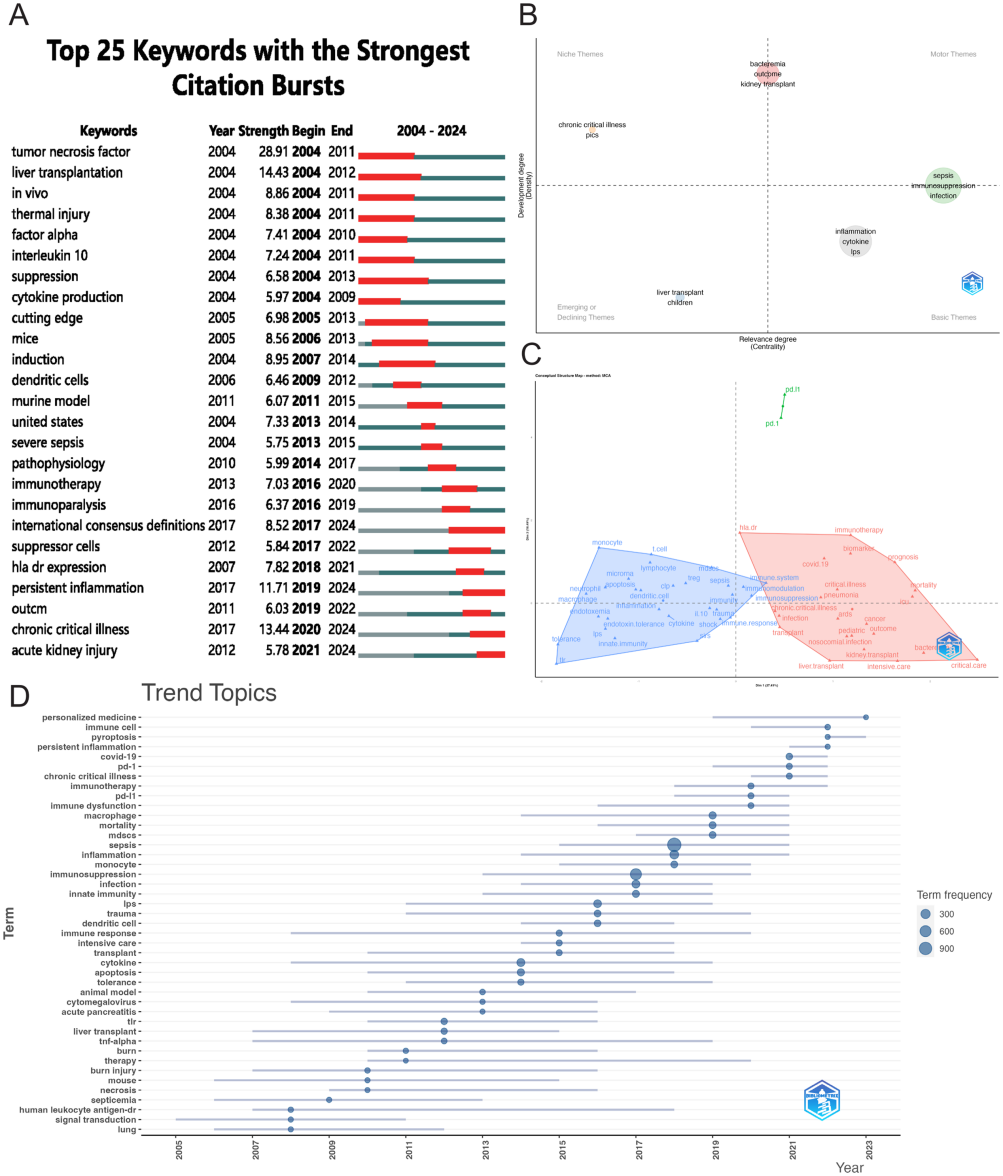
Visualizing hotspot changes. **(A)** The top 25 keywords with the strongest citation bursts. **(B)** Thematic map of research on sepsis immunosuppression. The horizontal axis denotes relevance, while the vertical axis represents developmental intensity. **(C)** Conceptual structure map. **(D)** Trend topics visualization.

Citation burst analysis is a valuable tool for identifying emerging research trends. Figure 10A presents the 25 most-cited keywords, with “tumor necrosis factor” showing the strongest citation burst (28.91), followed by “liver transplantation” (14.43) and “chronic critical illness” (13.44). The year 2004 marked the peak burst intensity, reflecting a pivotal period that spurred significant subsequent research. Four keywords continue to experience citation bursts: “international consensus definitions” (8.52), “persistent inflammation” (11.71), “chronic critical illness” (13.44), and “acute kidney injury” (5.78). Figure 10B presents a thematic map focusing on sepsis immunosuppression. The terms located in the upper-right quadrant (Quadrant I) correspond to the motor theme; the upper-left quadrant (Quadrant II) represents the niche theme; the lower-left quadrant (Quadrant III) contains emerging or declining themes, while the lower-right quadrant (Quadrant IV) encompasses basic themes. Figure 10C displays the linkages between keywords using multiple correspondence analysis, revealing two distinct clusters: the red cluster, focused on molecular-level research, and the blue cluster, oriented toward clinical applications. Additionally, Figure 10D further illustrates the evolving research focus, with recent emphasis on personalized medicine, immune cells, and emerging forms of cell death, such as pyroptosis.

**Figure 11:**
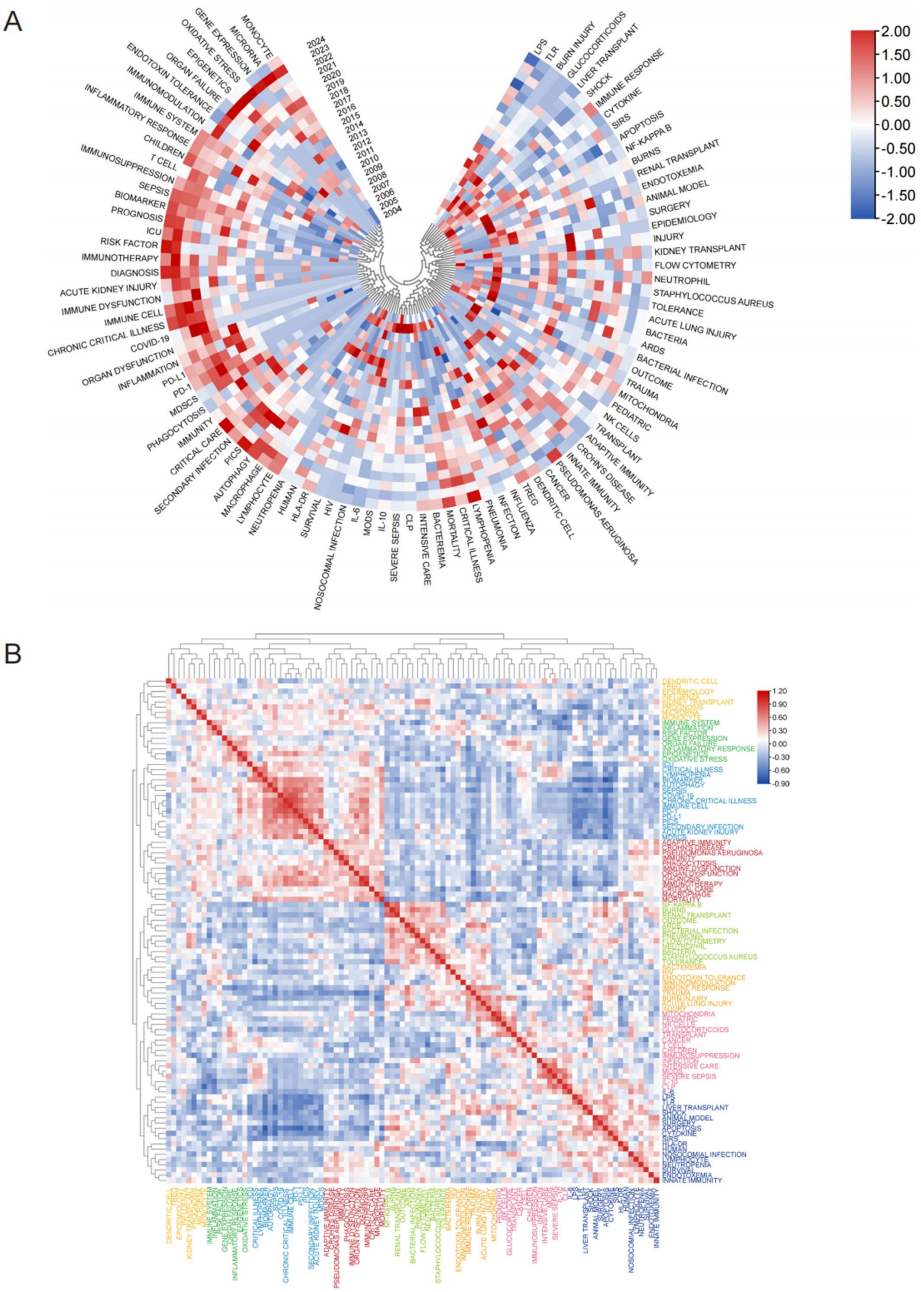
Analysis of hotspot shifts. **(A)** Annual heatmap depicting keyword popularity (2004–2024) in sepsis immunosuppression research, normalized by the annual citation proportion. **(B)** Keyword correlation heatmap. Keywords exhibiting high popularity within similar time periods are clustered together and color coded.

A heatmap (Figure 11A) was utilized to further visualize temporal trends in keyword prominence, highlighting recent research hotspots. Immune-related investigations primarily focused on immune regulation, immune dysfunction, immune system, and immunotherapy. At the molecular level, key themes included microRNA, gene expression, and epigenetics, while cellular research emphasized T cells, monocytes, MDSCs, dendritic cells, and various modes of cell death. Clinically, acute kidney injury (AKI), organ dysfunction, and prognostic assessment were major concerns. Terms related to critical illness, such as chronic critical illness, COVID-19, Critical Care, and secondary infection, were also frequently discussed. Figure 11B displays the popularity correlation of keywords. Keywords exhibiting high popularity during similar time periods were clustered into distinct groups and color-coded. The results reveal eight clusters: the yellow cluster (dendritic cells, Tregs, epigenetics, etc.), the green cluster (immune system, inflammation, risk factors, etc.), the blue cluster (ICU, critical illness, biomarkers, etc.), the red cluster (adaptive immunity, Crohn’s disease, *Pseudomonas aeruginosa*, etc.), the light-green cluster (NF-κB, burns, renal transplant, etc.), the pale-yellow cluster (bacteria, HIV, endotoxin tolerance, etc.), the pink cluster (mitochondria, pediatric, NK cells, etc.), and the dark-blue cluster (IL-6, LPS, TLR, etc.). This indicates that keywords within the same cluster exhibited comparable popularity during the same period.

## 4 Discussion

### 4.1 General analysis

The analysis reviewed a total of 3,082 articles authored by 16,545 researchers, covering the period from 2004 to 2024. The number of publications steadily increase, with 2018 marking the highest publication count. Despite a slight decline in publication numbers in 2022 and 2023, the overall trend has remained relatively stable. Interestingly, the citation frequency mirrored the number of publications, with a notable citation burst occurring in 2021.

In terms of geographic contributions, the United States led both in publication volume and citation frequency, underscoring its central role in this research area, particularly with the presence of several high-output institutions—four of which rank among the top ten. Although China ranked second in terms of publications, its citation count was only fifth. This discrepancy between publication volume and citation count could be attributed to two factors: China’s relatively recent engagement in sepsis immunosuppression research, which requires time for citations to accumulate, and the variability in the quality of Chinese publications. This highlights the importance of improving the quality of research alongside increasing publication output. The collaboration map further emphasized the United States as the primary research hub, with close collaborations with China and Germany, reinforcing the U.S.’s dominant position in the field.

The journals cited in the research are predominantly high-impact, indicating their significant value in sepsis immunosuppression studies. As shown in Table 3 and Figure 5A, Frontiers in Immunology published the most articles related to sepsis immunosuppression and ranked second in citation frequency. In contrast, Journal of Immunology published fewer than half the number of articles as Frontiers in Immunology but had a higher co-citation frequency, ranking first. Both journals are pivotal in advancing sepsis immunosuppression research, and future discoveries in this field are likely to be represented in them.

In terms of authorship, Monneret G from Hospices Civils de Lyon has contributed the most publications on sepsis immunosuppression, while Richard S. Hotchkiss, a professor at Washington University School of Medicine in St. Louis, United States, leads in co-citations, underscoring their leadership in the field. Professor Hotchkiss is a pioneer in elucidating the precise mechanisms of immunosuppression in sepsis. His research spans multiple facets of sepsis immunosuppression, including the roles of cytokines, regulation of apoptotic pathways, immune cell dysfunction, and the identification of novel therapeutic targets and approaches for sepsis treatment (7, 8, 20, 21). Additionally, these two leading scholars have collaborated extensively, publishing numerous reviews on sepsis immunosuppression and significantly advancing the field’s knowledge base.

The most frequently cited papers reflect their substantial academic influence. Table 5 lists the top 10 most-cited publications in sepsis-induced immunosuppression, while Figure 8 illustrates research hotspots identified through bibliometric clustering and lists 25 papers with citation bursts from 2004 to 2024. The most cited work, *Genomic responses in mouse models poorly mimic human inflammatory diseases*, holds the highest citation count, followed by *Sepsis-induced immunosuppression: from cellular dysfunctions to immunotherapy*, which ranks second, demonstrating their foundational impact in the field (7, 16).

A total of 5,006 keywords were analyzed, with “inflammation” emerging as the most frequently cited keyword, appearing 230 times. The top three keywords with the highest centrality— “shock,” “chronic critical illness,” and “immune response”— are all found in the dark blue cluster of Figure 9A, reflecting their clinical relevance to critically ill patients with sepsis. Figure 10D highlights the rising prominence of terms such as “personalized medicine,” “immune cell,” “pyroptosis,” and “persistent inflammation,” highlighting their current significance as focal points of investigation in sepsis-induced immunosuppression. Additionally, Figure 11A details recent research hotspots in sepsis-induced immunosuppression, with immunological aspects focusing on immunomodulation, immune dysfunction, and immunotherapy; molecular mechanisms primarily involving microRNA, gene expression, and epigenetic regulation; cellular components examining T cells, monocytes, MDSCs, dendritic cells, and cell death modalities; and clinical research emphasizing acute kidney injury, organ dysfunction, prognostic assessment, and critical conditions (e.g., COVID-19).

### 4.2 Research trends and hot topics

Bibliometric analysis of highly cited references and high-frequency keywords offers valuable insights into the evolving trends and core research themes within a field. Figures 8A, 10D illustrate the evolution of references and keyword trends from 2004 to 2024. The analysis reveals that, between 2004 and 2013, research primarily focused on two key areas. The first area was etiological exploration, with bibliometric evidence linking sepsis-related immunosuppression to underlying conditions such as “liver transplant” and “thermal injury.” Sepsis was also associated with factors like pathogen infections, immune status, age, genetic susceptibility, nosocomial infections, and antimicrobial resistance (4, 18). The second area of focus was the investigation of pathophysiological mechanisms, particularly the roles of inflammatory cytokines (e.g., “tumor necrosis factor”) and immune cells (e.g., “dendritic cells”). From 2013 onward, a paradigm shift occurred in the field, as research increasingly focused on immune mechanisms and immunotherapy. High-frequency keywords such as “immunotherapy” and “immune paralysis” emerged, reflecting this shift in focus. This change marks a conceptual evolution in the understanding of sepsis, transitioning from its traditional definition as “infection-induced systemic inflammatory response syndrome” to a recognition of the central role of immune response.

#### 4.2.1 Cell death in sepsis immunosuppression

As highlighted in Figures 8A, 10D, “pyroptosis” has emerged as a prominent keyword in recent research. Pyroptosis represents a genetically regulated cell death pathway executed through gasdermin pore formation, and is characterized by inflammasome activation, cytoplasmic membrane disintegration, and uncontrolled pro-inflammatory cytokine efflux (22). This process is primarily activated through two core pathways: the canonical pathway, where inflammasome-activated caspase-1 cleaves gasdermin-D (GSDMD), triggering pore formation and driving extracellular liberation of pro-inflammatory cytokines such as interleukin (IL)-18 and IL-1β (23–25); and the non-canonical pathway, in which lipopolysaccharide (LPS) directly binds and activates human caspase-4/5 (or murine caspase-11) (26), leading to GSDMD cleavage and subsequent pore formation by its N-terminal fragment in the cell membrane (27). Additionally, caspase-3 and caspase-8-dependent pathways can induce pyroptosis: chemotherapeutic agents trigger pyroptosis *via* caspase-3-mediated cleavage of GSDME (28), while Yersinia infection induces pyroptosis through caspase-8-mediated cleavage of GSDMD (29, 30). Research also suggests that granzyme B released by killer cells can directly cleave GSDME, mediating caspase-independent pyroptosis (31).

During sepsis, pyroptosis plays a dual role. On one hand, moderate pyroptosis aids in the clearance of intracellular pathogens. As demonstrated in Salmonella and Listeria infection models, pyroptosis lyses infected cells to prevent pathogen dissemination (32, 33). On the other hand, excessive pyroptosis contributes to a cytokine storm and immune cell exhaustion, resulting in immune dysregulation. For example, study shows that pyroptosis is a key driver of sepsis-induced secondary immune cell death (34), with its occurrence closely linked to lymphocytopenia and increased mortality in sepsis (35). Moreover, pyroptosis significantly contributes to sepsis-associated organ dysfunction. In LPS-induced septic shock, the caspy2-GSDMEb signaling pathway mediates lethal renal tubular injury, while the specific inhibitor Ac-FEID-CMK reduces the incidence of septic AKI and mortality in zebrafish (36). In the cecal ligation and puncture (CLP)-induced acute lung injury (ALI) model, inhibiting high mobility group box 1 (HMGB1) reduces caspase-11-dependent pyroptosis, thereby mitigating lung injury (37).

Significant advancements have been made in understanding the mechanisms of pyroptosis in sepsis. Research indicates that LPS-activated caspase-11 induces pyroptosis and severe inflammation through the pannexin-1 and P2X7 signaling pathways, positioning this axis as a potential therapeutic target for Gram-negative sepsis (38). Deng et al. demonstrated that hepatocyte-released HMGB1 transports extracellular LPS into the cytosol of macrophages and endothelial cells, activating caspase-11 and driving caspase-11-dependent pyroptosis, thereby contributing to sepsis lethality (39). Recent studies further suggest that extracellular vesicles carrying GSDMD pores integrate into neighboring cell membranes, propagating pyroptotic signals and initiating a cascade of inflammatory cell death, offering a novel mechanistic insight into systemic inflammatory dysregulation in sepsis (40). Additionally, a 2024 study revealed that palmitoyltransferase catalyzes the palmitoylation of full-length GSDMD, forming pores structurally similar to those generated by the GSDMD-N-terminal fragment, suggesting that GSDMD may trigger pyroptosis *via* palmitoylation as well as proteolytic activation (41).

In addition to pyroptosis, other forms of programmed cell death—such as apoptosis, autophagy, ferroptosis, and necroptosis—contribute significantly to the pathogenesis and progression of sepsis (42, 43). Ferroptosis, an iron-and reactive oxygen species (ROS)-dependent process driven by glutathione depletion, is characterized by lipid peroxide accumulation and plasma membrane rupture (44, 45). In sepsis, monocyte depletion is associated with ferroptosis, and ferroptosis in CD4 + T cells correlates with disease progression (46, 47). LPS-induced sepsis models show elevated ferroptosis markers (e.g., prostaglandin-endoperoxide synthase 2 (PTGS2), malondialdehyde (MDA), and lipid ROS), which are associated with increased disease severity, while ferroptosis inhibitors, such as dexrazoxane and ferrostatin-1, alleviate LPS-induced mitochondrial damage (48).

The role of apoptosis in sepsis is well-documented. In murine models of ALI induced by CLP or endotoxemia, neutrophil apoptosis in lung tissue was significantly reduced within 24 h post-injury (49). Inducing neutrophil apoptosis notably improved survival in septic mice (50). Unlike apoptosis, which preserves membrane integrity, necroptosis leads to plasma membrane rupture, releasing cellular contents such as damage-associated molecular patterns (DAMPs) and inflammatory cytokines, which amplify inflammation. Necroptosis is driven by the activation of mixed lineage kinase domain-like protein (MLKL), receptor-interacting protein kinase 1 (RIPK1), and RIPK3 (51). Necroptosis contributes to sepsis-associated organ injury (52, 53), and inhibiting key mediators like RIPK3 or RIPK1 alleviates systemic inflammation and organ damage in neonatal septic mice (54, 55). Autophagy, a lysosomal degradation process that recycles cellular components in response to stressors (particularly nutrient deprivation) (56), plays a critical role in sepsis. For example, T cell autophagy provides protective anti-apoptotic and immunosuppressive effects during sepsis (57).

Notably, these cell death pathways are interconnected rather than independent. For instance, necroptosis can act as an alternative death pathway when caspase-dependent apoptosis is inhibited (58). RIPK3 can also interact with caspase-8, switching cell fate from necroptosis to apoptosis (58). During sepsis, RIPK3-mediated necroptosis synergizes with GSDMD-induced pyroptosis, amplifying inflammatory signaling and exacerbating tissue damage. The recently proposed concept of “panoptosis” — a coordinated death mechanism involving pyroptosis, apoptosis, and necroptosis (59). And research demonstrates that Nerve injury-induced protein 1 (NINJ1) modulates thrombus formation and disseminated intravascular coagulation (DIC) in sepsis through regulating panoptosis and platelet activation (60).

Sepsis involves multiple interdependent cell death pathways forming an extensive crosstalk network, collectively driving its complex pathophysiology. Novel death modalities, such as “cuproptosis” (58), further elucidate sepsis complexity, with studies linking it to septic cardiotoxicity and sepsis-acquired muscle weakness (61, 62). A deeper understanding of these death pathways and their interactions could provide crucial insights for addressing sepsis-induced immune dysregulation and multi-organ injury.

#### 4.2.2 MDSCs in sepsis immunosuppression

Substantial evidence indicates that sepsis immunosuppression is characterized by death and dysfunction of immune effector cells and expansion of immunosuppressive cells (63). Abnormal expansion and hyperactivation of immunosuppressive cells—including myeloid-derived suppressor cells (MDSCs), regulatory T cells (Tregs), and M2 macrophages—represent a critical component of sepsis-induced immunosuppression (64). MDSCs are a heterogeneous population of bone marrow-derived cells, comprising pathologically activated neutrophils and monocytes, with potent immunosuppressive activity (65). Elevated MDSCs levels persist for at least 6 weeks in sepsis survivors post-infection (66), with this sustained expansion further linked to immunosuppression、 extended ICU stays and increased susceptibility to secondary infections (67–69).

MDSCs are primarily classified into two major subsets based on their phenotype and morphology: granulocytic/polymorphonuclear MDSCs (PMN-MDSCs) and monocytic MDSCs (M-MDSCs). Additionally, a group of myeloid precursor cells exhibiting MDSCs characteristics, referred to as “early MDSCs,” has been identified in humans. These early MDSCs, consisting mostly of myeloid progenitors and precursor cells, comprise less than 5% of the total MDSCs population (70). New markers have also been proposed to better characterize MDSCs. For instance, lectin-type oxidized low-density lipoprotein receptor-1 (LOX-1) demarcates human PMN-MDSCs (71), whereas S100 calcium-binding protein A9 (S100A9) serves as human M-MDSCs (72). Additionally, research indicates that monocyte-like granulocyte precursors can differentiate into PMN-MDSCs (73). Despite advances in high-resolution techniques like single-cell RNA sequencing, distinct phenotypic signatures to fully distinguish MDSCs and their subsets remain elusive, emphasizing the need for the discovery of more specific biomarkers.

The expansion of MDSCs is regulated by multiple mechanisms, including cytokines, growth factors, DAMPs (such as S100A8/A9), chemokines (e.g., CXC motif chemokine ligand (CXCL) 1, CXCL2), signaling pathways, and epigenetic modifications. Cytokines such as granulocyte-macrophage colony stimulating factor (GM-CSF), G-CSF, IL-6, and IL-10 promote the differentiation of bone marrow progenitors into MDSCs (74, 75). S100A8/A9 interact with carboxylated N-glycans on glycoprotein receptors on the MDSCs surface, activating the nuclear factor-κB (NF-κB) pathway and facilitating MDSCs accumulation (76). Growth factors like vascular endothelial growth factor (VEGF) and hepatocyte growth factor (HGF) stimulate MDSCs expansion and inhibit their maturation *via* activation of the signal transducer and activator of transcription 3 (STAT3) pathway (77). Toll-like receptor 4 (TLR4) and myeloiddifferentiationfactor88 (MyD88) are also involved in sepsis-induced PMN-MDSCs proliferation (78). Epigenetic modifications, such as H3K27me3 demethylation and H3K4me3 activation, dynamically regulate the transcription of the long non-coding RNA Hotairm1 gene, thereby programming MDSCs function and driving their pathological accumulation during late-stage sepsis (79).

The immunosuppressive activity of MDSCs in sepsis is regulated by several mechanisms. First, aberrant activation of sepsis-associated signaling pathways modulates their immunosuppressive function. Cytokines such as tumor necrosis factor (TNF) and IL-1*β* enhance MDSCs suppressive activity by activating nuclear NF-κB (80). Pro-inflammatory factors, including prostaglandin E2 (PGE2), LPS, IL-1β, and Interferon-*γ* (IFN-γ), activate cyclooxygenase-2 (COX2), which correlates with the expression of MDSCs-associated inhibitory factors like IL-4Rα, nitric oxide synthase 2 (NOS2), and IL-10 (81). Additionally, a galectin-1 (GAL1)-driven glycosylation-dependent program activates the STAT3 signaling pathway through binding to the CD18-CD11b-CD177 receptor complex on MDSCs, modulating their immunosuppressive and angiogenic functions (82). The endoplasmic reticulum stress response also plays a role in regulating MDSCs function. ROS and peroxynitrite promote the upregulation of C/EBP homologous protein (CHOP) expression in MDSCs, driving their accumulation and immunosuppressive activity (83). Moreover, metabolic reprogramming is critical for MDSCs survival, accumulation, and immunosuppressive function in sepsis. For instance, the acidic microenvironment induced by lactate production and glucose deprivation during sepsis forces MDSCs to rely on glycolysis for energy supply (84).

MDSCs primarily function to inhibit immune responses. They suppress both adaptive and innate immunity *via* multiple mechanisms, notably exerting strong inhibition on T cells (85, 86). For instance, MDSCs can secrete immunosuppressive cytokines, such as IL-10 and transforming growth factor-β (TGF-β) to suppress T-cell proliferation, activation, trafficking, and effector functions (81, 87, 88), while concurrently upregulating programmed death-ligand 1 (PD-L1) expression to attenuate T-cell responsiveness (89). Conversely, these cells promote Tregs differentiation and expansion (90). Furthermore, they reduce the cytotoxicity of natural killer (NK) cells by decreasing the expression of natural killer group 2 member D (NKG2D) and IFN-γ receptors (91). However, evidence suggests MDSCs may also play protective roles in specific contexts. MDSCs migration during the late phase of sepsis may contribute to the long-term survival of patients. Supporting this, taurodeoxycholic acid (TDCA) ameliorates sepsis in mice by increasing MDSCs abundance (92), while neutralizing GR-1 to deplete MDSCs in CLP mice decreased survival relative to sham controls (93). Furthermore, recent reports indicate that M-MDSCs-dominated immunosuppression may confer protection during the early stage of critical illness by modulating T and NK cell function (94). Additionally, the transient appearance of MDSCs in early life provides significant protection against neonatal inflammatory diseases. For example, the bile acid receptor farnesoid X receptor (FXR) protects against neonatal sepsis by enhancing the immunosuppressive function of MDSCs (95). Similarly, adoptive transfer of MDSCs has been shown to mitigate neonatal inflammation induced by maternal circadian rhythm disruption (96). Finally, activation of PMN-MDSCs during pregnancy has been reported to support fetal growth (97).

The expansion and activation of MDSCs are closely linked to immunosuppression and prognosis in sepsis. This population of cells holds promise as both a biomarker and a therapeutic target for sepsis. However, their heterogeneity presents significant challenges. Current methods for phenotypic and functional characterization lack precision, and the mechanisms governing their expansion and immunosuppressive molecular networks are not fully understood. Future research should focus on elucidating the developmental trajectory of MDSCs in sepsis, identifying specific biomarkers, and developing targeted therapies against MDSCs-associated immune checkpoints, which could lead to therapeutic breakthroughs in sepsis immunotherapy.

#### 4.2.3 Clinical treatment of immunosuppression in sepsis

Traditional therapeutic strategies for sepsis primarily focus on early antimicrobial treatment and supportive care (98). While antibiotics and supportive measures have reduced early mortality, many survivors ultimately succumb to persistent, recurrent, or secondary infections. In this phase, patients display sustained low-grade inflammation and immunosuppression, shifting the research emphasis from anti-inflammatory strategies to therapies targeting dysregulated host immunity (63).

Immune checkpoint molecules, particularly programmed death protein 1/programmed death ligand 1 (PD-1/PD-L1), have emerged as promising therapeutic targets. Studies show that in the CLP-induced sepsis model, PD-1-deficient mice have improved survival, while PD-L1 blockade improves outcomes by inhibiting lymphocyte apoptosis and restoring macrophage function (81, 99). Recent findings highlight that the itaconate derivative 4-octyl itaconate (OI) significantly reduces mortality in septic mice by mitigating tissue/organ damage, decreasing pro-inflammatory cytokines, and increasing anti-inflammatory cytokine expression (100). Restoring immune function is a critical strategy for treating sepsis-induced immunosuppression. During sepsis, IFN-*γ* production is suppressed, and its restoration improves survival in murine models (101). Immune stimulants, such as IFN-γ and GM-CSF, have entered clinical trials, and artesunate has been shown to reverse secondary infections in CLP-induced immunocompromised septic mice (102).

However, the heterogeneity of immune phenotypes among septic patients—driven by factors such as pathogen type (Gram-positive/negative bacteria, fungi), infection site (abdomen, lung, bloodstream), and host variables (age, genetics)—limits the efficacy of conventional immunomodulatory drugs. This highlights the need for personalized immunotherapy and precision medicine, which have become central research priorities. Future efforts should focus on dynamically analyzing the pathophysiological mechanisms across different disease stages to better understand this heterogeneity and enable precision treatment. Cutting-edge technologies such as single-cell transcriptomics, multi-omics integration (transcriptomics, proteomics, metabolomics), and artificial intelligence (AI)-driven machine learning are revolutionizing our ability to decipher the complexity of sepsis, paving the way for individualized diagnostic and therapeutic strategies.

### 4.3 Limitations

This study has several limitations. Firstly, the data used for analysis were sourced from the WoSCC database, with a language restriction to English. This may have excluded relevant publications from other databases or in other languages, potentially limiting the comprehensiveness of the study. Additionally, variability in the quality of the collected articles could introduce bias, affecting the reliability of the analysis. Moreover, the cluster analysis conducted using CiteSpace and VOSviewer may be susceptible to inconsistencies in terms extracted from literature titles, abstracts, and keywords. Terms with similar meanings may not always be consistently grouped, which could impact the results. These limitations should be considered when interpreting the findings.

## 5 Conclusion

Despite these limitations, research data from 2004 to 2024 offer valuable insights into sepsis immunosuppression. Notable contributors, such as Guillaume Monneret and Richard S. Hotchkiss, have significantly advanced the field. The United States leads in the number of published articles, reflecting its active involvement in sepsis immunosuppression research. The findings highlight the importance of further research in sepsis immunosuppression, as well as the potential for continued collaboration between countries like the U.S., China, and Germany. Journals such as Frontiers in Immunology have published a substantial number of articles in the field, while others like the Journal of Immunology boast a higher co-citation frequency. These journals play a pivotal role in disseminating research and advancing knowledge in the field. Immune cells, cell death, and personalized clinical treatments have emerged as hot topics in this field. Overall, this study provides valuable insights into research trends and topics in sepsis immunosuppression, and paves the way for further exploration and collaboration in this critical area of research.

## Data Availability

The original contributions presented in the study are included in the article/supplementary material, further inquiries can be directed to the corresponding authors.

## References

[1] Mayr FB Yende S Angus DC. Epidemiology of severe sepsis. Virulence. (2014) 5:4–11. doi: 10.4161/viru.27372, PMID: 24335434 PMC3916382

[2] Patil NK Guo Y Luan L Sherwood ER. Targeting immune cell checkpoints during Sepsis. Int J Mol Sci. (2017) 18:2413. doi: 10.3390/ijms18112413, PMID: 29135922 PMC5713381

[3] Singer M Deutschman CS Seymour CW Shankar-Hari M Annane D Bauer M . The third international consensus definitions for Sepsis and septic shock (Sepsis-3). JAMA. (2016) 315:801–10. doi: 10.1001/jama.2016.0287, PMID: 26903338 PMC4968574

[4] Cecconi M Evans L Levy M Rhodes A. Sepsis and septic shock. Lancet. (2018) 392:75–87. doi: 10.1016/S0140-6736(18)30696-2 29937192

[5] SepNet Critical Care Trials Group. Incidence of severe sepsis and septic shock in German intensive care units: the prospective, multicentre INSEP study. Intensive Care Med. (2016) 42:1980–9. doi: 10.1007/s00134-016-4504-3, PMID: 27686355

[6] Vincent J-L Marshall JC Namendys-Silva SA François B Martin-Loeches I Lipman J . Assessment of the worldwide burden of critical illness: the intensive care over nations (ICON) audit. Lancet Respir Med. (2014) 2:380–6. doi: 10.1016/S2213-2600(14)70061-X, PMID: 24740011

[7] Hotchkiss RS Monneret G Payen D. Sepsis-induced immunosuppression: from cellular dysfunctions to immunotherapy. Nat Rev Immunol. (2013) 13:862–74. doi: 10.1038/nri3552, PMID: 24232462 PMC4077177

[8] Hotchkiss RS Opal S. Immunotherapy for sepsis--a new approach against an ancient foe. N Engl J Med. (2010) 363:87–9. doi: 10.1056/NEJMcibr1004371, PMID: 20592301 PMC4136660

[9] Hotchkiss RS Monneret G Payen D. Immunosuppression in sepsis: a novel understanding of the disorder and a new therapeutic approach. Lancet Infect Dis. (2013) 13:260–8. doi: 10.1016/S1473-3099(13)70001-X, PMID: 23427891 PMC3798159

[10] Gotts JE Matthay MA. Sepsis: pathophysiology and clinical management. BMJ. (2016) 353:i1585. doi: 10.1136/bmj.i1585, PMID: 27217054

[11] Hicks D Wouters P Waltman L de Rijcke S Rafols I. Bibliometrics: the Leiden manifesto for research metrics. Nature. (2015) 520:429–31. doi: 10.1038/520429a, PMID: 25903611

[12] Hassan W Duarte AE. Bibliometric analysis: a few suggestions. Curr Probl Cardiol. (2024) 49:102640. doi: 10.1016/j.cpcardiol.2024.102640, PMID: 38740289

[13] Chen CM. CiteSpace II: detecting and visualizing emerging trends and transient patterns in scientific literature. J Am Soc Inf Sci Technol. (2006) 57:359–77. doi: 10.1002/asi.20317

[14] Synnestvedt MB Chen C Holmes JH. CiteSpace II: visualization and knowledge discovery in bibliographic databases. AMIA Annu Symp Proc. (2005) 2005:724–8. 16779135 PMC1560567

[15] van Eck NJ Waltman L. Software survey: VOSviewer, a computer program for bibliometric mapping. Scientometrics. (2010) 84:523–38. doi: 10.1007/s11192-009-0146-3, PMID: 20585380 PMC2883932

[16] Seok J Warren HS Cuenca AG Mindrinos MN Baker HV Xu W . Genomic responses in mouse models poorly mimic human inflammatory diseases. Proc Natl Acad Sci USA. (2013) 110:3507–12. doi: 10.1073/pnas.1222878110, PMID: 23401516 PMC3587220

[17] Venet F Monneret G. Advances in the understanding and treatment of sepsis-induced immunosuppression. Nat Rev Nephrol. (2018) 14:121–37. doi: 10.1038/nrneph.2017.165, PMID: 29225343

[18] Rudd KE Johnson SC Agesa KM Shackelford KA Tsoi D Kievlan DR . Global, regional, and national sepsis incidence and mortality, 1990-2017: analysis for the global burden of disease study. Lancet. (2020) 395:200–11. doi: 10.1016/S0140-6736(19)32989-7, PMID: 31954465 PMC6970225

[19] van der Poll T Shankar-Hari M Wiersinga WJ. The immunology of sepsis. Immunity. (2021) 54:2450–64. doi: 10.1016/j.immuni.2021.10.012, PMID: 34758337

[20] Hotchkiss RS Tinsley KW Swanson PE Grayson MH Osborne DF Wagner TH . Depletion of dendritic cells, but not macrophages, in patients with sepsis. J Immunol. (2002) 168:2493–500. doi: 10.4049/jimmunol.168.5.2493, PMID: 11859143

[21] Hotchkiss RS Tinsley KW Swanson PE Schmieg RE Jr Hui JJ Chang KC . Sepsis-induced apoptosis causes progressive profound depletion of B and CD4+ T lymphocytes in humans. J Immunol. (2001) 166:6952–63. doi: 10.4049/jimmunol.166.11.6952, PMID: 11359857

[22] Broz P. Immunology: caspase target drives pyroptosis. Nature. (2015) 526:642–3. doi: 10.1038/nature15632, PMID: 26375000

[23] Cookson BT Brennan MA. Pro-inflammatory programmed cell death. Trends Microbiol. (2001) 9:113–4. doi: 10.1016/s0966-842x(00)01936-3 11303500

[24] He Y Amer AO. Microbial modulation of host apoptosis and pyroptosis. Front Cell Infect Microbiol. (2014) 4:83. doi: 10.3389/fcimb.2014.00083, PMID: 24995165 PMC4062964

[25] Jackson DN Theiss AL. Gut bacteria signaling to mitochondria in intestinal inflammation and cancer. Gut Microbes. (2020) 11:285–304. doi: 10.1080/19490976.2019.1592421 30913966 PMC7524274

[26] Shi J Zhao Y Wang Y Gao W Ding J Li P . Inflammatory caspases are innate immune receptors for intracellular LPS. Nature. (2014) 514:187–92. doi: 10.1038/nature13683 25119034

[27] Chu LH Indramohan M Ratsimandresy RA Gangopadhyay A Morris EP Monack DM . The oxidized phospholipid oxPAPC protects from septic shock by targeting the non-canonical inflammasome in macrophages. Nat Commun. (2018) 9:996. doi: 10.1038/s41467-018-03409-3, PMID: 29520027 PMC5843631

[28] Wang Y Gao W Shi X Ding J Liu W He H . Chemotherapy drugs induce pyroptosis through caspase-3 cleavage of a gasdermin. Nature. (2017) 547:99–103. doi: 10.1038/nature22393, PMID: 28459430

[29] Sarhan J Liu BC Muendlein HI Li P Nilson R Tang AY . Caspase-8 induces cleavage of gasdermin D to elicit pyroptosis during Yersinia infection. Proc Natl Acad Sci USA. (2018) 115:E10888–97. doi: 10.1073/pnas.1809548115, PMID: 30381458 PMC6243247

[30] Orning P Weng D Starheim K Ratner D Best Z Lee B . Pathogen blockade of TAK1 triggers caspase-8-dependent cleavage of gasdermin D and cell death. Science. (2018) 362:1064–9. doi: 10.1126/science.aau2818, PMID: 30361383 PMC6522129

[31] Zhang Z Zhang Y Xia S Kong Q Li S Liu X . Gasdermin E suppresses tumour growth by activating anti-tumour immunity. Nature. (2020) 579:415–20. doi: 10.1038/s41586-020-2071-9, PMID: 32188940 PMC7123794

[32] Liu X Zhang Z Ruan J Pan Y Magupalli VG Wu H . Inflammasome-activated gasdermin D causes pyroptosis by forming membrane pores. Nature. (2016) 535:153–8. doi: 10.1038/nature18629, PMID: 27383986 PMC5539988

[33] Jorgensen I Rayamajhi M Miao EA. Programmed cell death as a defence against infection. Nat Rev Immunol. (2017) 17:151–64. doi: 10.1038/nri.2016.147, PMID: 28138137 PMC5328506

[34] Li F Han X Wu C He J Liu H Li S . Evaluation of immune and pyroptosis status in a model of sepsis-induced secondary pneumonia. Int Immunopharmacol. (2024) 140:112835. doi: 10.1016/j.intimp.2024.112835, PMID: 39088917

[35] Guo R Zhao G Bai G Chen J Han W Cui N . Depletion of mTOR ameliorates CD4+ T cell pyroptosis by promoting autophagy activity in septic mice. Int Immunopharmacol. (2023) 124:110964. doi: 10.1016/j.intimp.2023.110964, PMID: 37738689

[36] Wang Z Gu Z Hou Q Chen W Mu D Zhang Y . Zebrafish GSDMEb cleavage-gated Pyroptosis drives septic acute kidney injury in vivo. J Immunol. (2020) 204:1929–42. doi: 10.4049/jimmunol.1901456, PMID: 32111733

[37] Xie K Chen YQ Chai YS Lin SH Wang CJ Xu F. HMGB1 suppress the expression of IL-35 by regulating naïve CD4+ T cell differentiation and aggravating Caspase-11-dependent pyroptosis in acute lung injury. Int Immunopharmacol. (2021) 91:107295. doi: 10.1016/j.intimp.2020.107295, PMID: 33360086

[38] Yang D He Y Muñoz-Planillo R Liu Q Núñez G. Caspase-11 requires the Pannexin-1 channel and the purinergic P2X7 pore to mediate Pyroptosis and Endotoxic shock. Immunity. (2015) 43:923–32. doi: 10.1016/j.immuni.2015.10.009, PMID: 26572062 PMC4795157

[39] Deng M Tang Y Li W Wang X Zhang R Zhang X . The endotoxin delivery protein HMGB1 mediates Caspase-11-dependent lethality in Sepsis. Immunity. (2018) 49:740–753.e7. doi: 10.1016/j.immuni.2018.08.016, PMID: 30314759 PMC6300139

[40] Wright SS Kumari P Fraile-Ágreda V Wang C Shivcharan S Kappelhoff S . Transplantation of gasdermin pores by extracellular vesicles propagates pyroptosis to bystander cells. Cell. (2025) 188:280–291.e17. doi: 10.1016/j.cell.2024.11.018, PMID: 39742811 PMC12272064

[41] Du G Healy LB David L Walker C El-Baba TJ Lutomski CA . ROS-dependent S-palmitoylation activates cleaved and intact gasdermin D. Nature. (2024) 630:437–46. doi: 10.1038/s41586-024-07373-5, PMID: 38599239 PMC11283288

[42] Zhu H Santo A Jia Z Robert Li Y. GPx4 in bacterial infection and Polymicrobial Sepsis: Involvement of Ferroptosis and Pyroptosis. React Oxyg Species (Apex). (2019) 7:154–60. doi: 10.20455/ros.2019.835, PMID: 31106276 PMC6519466

[43] Du Y Zhong Y Ding R Wang X Xia F Zhang Q . New insights of necroptosis and immune infiltration in sepsis-induced myocardial dysfunction from bioinformatics analysis through RNA-seq in mice. Front Cell Infect Microbiol. (2022) 12:1068324. doi: 10.3389/fcimb.2022.1068324, PMID: 36619743 PMC9811394

[44] Dixon SJ Lemberg KM Lamprecht MR Skouta R Zaitsev EM Gleason CE . Ferroptosis: an iron-dependent form of nonapoptotic cell death. Cell. (2012) 149:1060–72. doi: 10.1016/j.cell.2012.03.042, PMID: 22632970 PMC3367386

[45] Tang D Kang R Berghe TV Vandenabeele P Kroemer G. The molecular machinery of regulated cell death. Cell Res. (2019) 29:347–64. doi: 10.1038/s41422-019-0164-5, PMID: 30948788 PMC6796845

[46] Kong Z Cai S Xie W Chen J Xie J Yang F . CD4 + T cells ferroptosis is associated with the development of sepsis in severe polytrauma patients. Int Immunopharmacol. (2024) 127:111377. doi: 10.1016/j.intimp.2023.111377, PMID: 38104369

[47] Zhang T Wang S Hua D Shi X Deng H Jin S . Identification of ZIP8-induced ferroptosis as a major type of cell death in monocytes under sepsis conditions. Redox Biol. (2024) 69:102985. doi: 10.1016/j.redox.2023.102985, PMID: 38103342 PMC10764267

[48] Li N Wang W Zhou H Wu Q Duan M Liu C . Ferritinophagy-mediated ferroptosis is involved in sepsis-induced cardiac injury. Free Radic Biol Med. (2020) 160:303–18. doi: 10.1016/j.freeradbiomed.2020.08.009, PMID: 32846217

[49] Wang J-F Wang YP Xie J Zhao ZZ Gupta S Guo Y . Upregulated PD-L1 delays human neutrophil apoptosis and promotes lung injury in an experimental mouse model of sepsis. Blood. (2021) 138:806–10. doi: 10.1182/blood.2020009417, PMID: 34473230

[50] Zhang CY Dong X Gao J Lin W Liu Z Wang Z. Nanoparticle-induced neutrophil apoptosis increases survival in sepsis and alleviates neurological damage in stroke. Sci Adv. (2019) 5:eaax7964. doi: 10.1126/sciadv.aax7964, PMID: 31723603 PMC6834394

[51] Pasparakis M Vandenabeele P. Necroptosis and its role in inflammation. Nature. (2015) 517:311–20. doi: 10.1038/nature14191, PMID: 25592536

[52] Sureshbabu A Patino E Ma KC Laursen K Finkelsztein EJ Akchurin O . RIPK3 promotes sepsis-induced acute kidney injury via mitochondrial dysfunction. JCI Insight. (2018) 3:e98411. doi: 10.1172/jci.insight.98411, PMID: 29875323 PMC6124406

[53] Xu Q Guo J Li X Wang Y Wang D Xiao K . Necroptosis underlies hepatic damage in a piglet model of lipopolysaccharide-induced Sepsis. Front Immunol. (2021) 12:633830. doi: 10.3389/fimmu.2021.633830, PMID: 33777021 PMC7994362

[54] Bolognese AC Yang WL Hansen LW Denning NL Nicastro JM Coppa GF . Inhibition of necroptosis attenuates lung injury and improves survival in neonatal sepsis. Surgery. (2018) 164:110–6. doi: 10.1016/j.surg.2018.02.017, PMID: 29709367 PMC6204110

[55] Hansen LW Jacob A Yang WL Bolognese AC Prince J Nicastro JM . Deficiency of receptor-interacting protein kinase 3 (RIPK3) attenuates inflammation and organ injury in neonatal sepsis. J Pediatr Surg. (2018) 53:1699–705. doi: 10.1016/j.jpedsurg.2017.11.054, PMID: 29248164 PMC5966335

[56] Iba T Helms J Maier CL Ferrer R Levy JH. Autophagy and autophagic cell death in sepsis: friend or foe? J Intensive Care. (2024) 12:41. doi: 10.1186/s40560-024-00754-y, PMID: 39449054 PMC11520123

[57] Oami T Watanabe E Hatano M Sunahara S Fujimura L Sakamoto A . Suppression of T cell autophagy results in decreased viability and function of T cells through accelerated apoptosis in a murine Sepsis model. Crit Care Med. (2017) 45:e77–85. doi: 10.1097/CCM.0000000000002016, PMID: 27618275 PMC5364514

[58] Wang W Gui H Wei X Liu H Wang X. Cuproptosis to Cuproptosis-like: therapeutic strategies for bacterial infection. Adv Mater. (2025):e2506119. doi: 10.1002/adma.202506119, PMID: 40538266

[59] Wang Y Kanneganti T-D. From pyroptosis, apoptosis and necroptosis to PANoptosis: a mechanistic compendium of programmed cell death pathways. Comput Struct Biotechnol J. (2021) 19:4641–57. doi: 10.1016/j.csbj.2021.07.038, PMID: 34504660 PMC8405902

[60] Zhou X Yu X Wan C Li F Wang Y Zhang K . NINJ1 regulates platelet activation and PANoptosis in septic disseminated intravascular coagulation. Int J Mol Sci. (2023) 24:4168. doi: 10.3390/ijms24044168, PMID: 36835580 PMC9958814

[61] Yan J Li Z Li Y Zhang Y. Sepsis induced cardiotoxicity by promoting cardiomyocyte cuproptosis. Biochem Biophys Res Commun. (2024) 690:149245. doi: 10.1016/j.bbrc.2023.149245, PMID: 38006800

[62] Yang L Xie L Li M Miao Y Yang J Chen S . Potential relationship between cuproptosis and sepsis-acquired weakness: an intermediate role for mitochondria. Front Physiol. (2025) 16:1520669. doi: 10.3389/fphys.2025.1520669, PMID: 40182687 PMC11965645

[63] Gao X Cai S Li X Wu G. Sepsis-induced immunosuppression: mechanisms, biomarkers and immunotherapy. Front Immunol. (2025) 16:1577105. doi: 10.3389/fimmu.2025.1577105, PMID: 40364841 PMC12069044

[64] Liu D Huang SY Sun JH Zhang HC Cai QL Gao C . Sepsis-induced immunosuppression: mechanisms, diagnosis and current treatment options. Mil Med Res. (2022) 9:56. doi: 10.1186/s40779-022-00422-y, PMID: 36209190 PMC9547753

[65] Ost M Singh A Peschel A Mehling R Rieber N Hartl D. Myeloid-derived suppressor cells in bacterial infections. Front Cell Infect Microbiol. (2016) 6:37. doi: 10.3389/fcimb.2016.00037, PMID: 27066459 PMC4814452

[66] Hollen MK Stortz JA Darden D Dirain ML Nacionales DC Hawkins RB . Myeloid-derived suppressor cell function and epigenetic expression evolves over time after surgical sepsis. Crit Care. (2019) 23:355. doi: 10.1186/s13054-019-2628-x, PMID: 31722736 PMC6854728

[67] Mathias B Delmas AL Ozrazgat-Baslanti T Vanzant EL Szpila BE Mohr AM . Human myeloid-derived suppressor cells are associated with chronic immune suppression after severe Sepsis/septic shock. Ann Surg. (2017) 265:827–34. doi: 10.1097/SLA.0000000000001783, PMID: 27163951 PMC5102824

[68] Uhel F Azzaoui I Grégoire M Pangault C Dulong J Tadié JM . Early expansion of circulating granulocytic myeloid-derived suppressor cells predicts development of nosocomial infections in patients with Sepsis. Am J Respir Crit Care Med. (2017) 196:315–27. doi: 10.1164/rccm.201606-1143OC, PMID: 28146645

[69] Veglia F Sanseviero E Gabrilovich DI. Myeloid-derived suppressor cells in the era of increasing myeloid cell diversity. Nat Rev Immunol. (2021) 21:485–98. doi: 10.1038/s41577-020-00490-y, PMID: 33526920 PMC7849958

[70] Bronte V Brandau S Chen SH Colombo MP Frey AB Greten TF . Recommendations for myeloid-derived suppressor cell nomenclature and characterization standards. Nat Commun. (2016) 7:12150. doi: 10.1038/ncomms12150, PMID: 27381735 PMC4935811

[71] Condamine T Dominguez GA Youn JI Kossenkov AV Mony S Alicea-Torres K . Lectin-type oxidized LDL receptor-1 distinguishes population of human polymorphonuclear myeloid-derived suppressor cells in cancer patients. Sci Immunol. (2016) 1:aaf8943. doi: 10.1126/sciimmunol.aaf8943, PMID: 28417112 PMC5391495

[72] Zhao F Hoechst B Duffy A Gamrekelashvili J Fioravanti S Manns MP . S100A9 a new marker for monocytic human myeloid-derived suppressor cells. Immunology. (2012) 136:176–83. doi: 10.1111/j.1365-2567.2012.03566.x, PMID: 22304731 PMC3403264

[73] Mastio J Condamine T Dominguez G Kossenkov AV Donthireddy L Veglia F . Identification of monocyte-like precursors of granulocytes in cancer as a mechanism for accumulation of PMN-MDSCs. J Exp Med. (2019) 216:2150–69. doi: 10.1084/jem.20181952, PMID: 31239386 PMC6719429

[74] Patel JJ Rosenthal MD McClave SA Martindale RG. Tempering the clinical effects of early myeloid-derived suppressor cell expansion in severe Sepsis and septic shock. Am J Respir Crit Care Med. (2018) 197:677–8. doi: 10.1164/rccm.201708-1701LE, PMID: 28984471

[75] Schrijver IT Théroude C Roger T. Myeloid-derived suppressor cells in Sepsis. Front Immunol. (2019) 10:327. doi: 10.3389/fimmu.2019.00327, PMID: 30873175 PMC6400980

[76] Sinha P Okoro C Foell D Freeze HH Ostrand-Rosenberg S Srikrishna G. Proinflammatory S100 proteins regulate the accumulation of myeloid-derived suppressor cells. J Immunol. (2008) 181:4666–75. doi: 10.4049/jimmunol.181.7.4666, PMID: 18802069 PMC2810501

[77] Melani C Chiodoni C Forni G Colombo MP. Myeloid cell expansion elicited by the progression of spontaneous mammary carcinomas in c-erbB-2 transgenic BALB/c mice suppresses immune reactivity. Blood. (2003) 102:2138–45. doi: 10.1182/blood-2003-01-0190, PMID: 12750171

[78] Llitjos J-F Auffray C Alby-Laurent F Rousseau C Merdji H Bonilla N . Sepsis-induced expansion of granulocytic myeloid-derived suppressor cells promotes tumour growth through toll-like receptor 4. J Pathol. (2016) 239:473–83. doi: 10.1002/path.4744 27178223

[79] Bah I Youssef D Yao ZQ McCall CE el Gazzar M. Inhibiting KDM6A demethylase represses Long non-coding RNA Hotairm1 transcription in MDSC during Sepsis. Front Immunol. (2022) 13:823660. doi: 10.3389/fimmu.2022.823660, PMID: 35185915 PMC8851568

[80] Condamine T Mastio J Gabrilovich DI. Transcriptional regulation of myeloid-derived suppressor cells. J Leukoc Biol. (2015) 98:913–22. doi: 10.1189/jlb.4RI0515-204R, PMID: 26337512 PMC4661041

[81] Zhang W Fang X Gao C Song C He Y Zhou T . MDSCs in sepsis-induced immunosuppression and its potential therapeutic targets. Cytokine Growth Factor Rev. (2023) 69:90–103. doi: 10.1016/j.cytogfr.2022.07.007, PMID: 35927154

[82] Blidner AG Bach CA García PA Merlo JP Cagnoni AJ Bannoud N . Glycosylation-driven programs coordinate immunoregulatory and pro-angiogenic functions of myeloid-derived suppressor cells. Immunity. (2025) 58:1553–1571.e8. doi: 10.1016/j.immuni.2025.04.027, PMID: 40381622

[83] Thevenot PT Sierra RA Raber PL al-Khami AA Trillo-Tinoco J Zarreii P . The stress-response sensor chop regulates the function and accumulation of myeloid-derived suppressor cells in tumors. Immunity. (2014) 41:389–401. doi: 10.1016/j.immuni.2014.08.015, PMID: 25238096 PMC4171711

[84] Jian S-L Chen WW Su YC Su YW Chuang TH Hsu SC . Glycolysis regulates the expansion of myeloid-derived suppressor cells in tumor-bearing hosts through prevention of ROS-mediated apoptosis. Cell Death Dis. (2017) 8:e2779. doi: 10.1038/cddis.2017.192, PMID: 28492541 PMC5520713

[85] Marigo I Bosio E Solito S Mesa C Fernandez A Dolcetti L . Tumor-induced tolerance and immune suppression depend on the C/EBPbeta transcription factor. Immunity. (2010) 32:790–802. doi: 10.1016/j.immuni.2010.05.010, PMID: 20605485

[86] Sinha P Clements VK Bunt SK Albelda SM Ostrand-Rosenberg S. Cross-talk between myeloid-derived suppressor cells and macrophages subverts tumor immunity toward a type 2 response. J Immunol. (2007) 179:977–83. doi: 10.4049/jimmunol.179.2.977, PMID: 17617589

[87] Cuenca AG Delano MJ Kelly-Scumpia KM Moreno C Scumpia PO Laface DM . A paradoxical role for myeloid-derived suppressor cells in sepsis and trauma. Mol Med. (2011) 17:281–92. doi: 10.2119/molmed.2010.00178 21085745 PMC3060988

[88] Goldmann O Nwofor OV Chen Q Medina E. Mechanisms underlying immunosuppression by regulatory cells. Front Immunol. (2024) 15:1328193. doi: 10.3389/fimmu.2024.1328193, PMID: 38380317 PMC10876998

[89] Hou A Hou K Huang Q Lei Y Chen W. Targeting myeloid-derived suppressor cell, a promising strategy to overcome resistance to immune checkpoint inhibitors. Front Immunol. (2020) 11:783. doi: 10.3389/fimmu.2020.00783, PMID: 32508809 PMC7249937

[90] Wang H Zhou F Qin W Yang Y Li X Liu R. Metabolic regulation of myeloid-derived suppressor cells in tumor immune microenvironment: targets and therapeutic strategies. Theranostics. (2025) 15:2159–84. doi: 10.7150/thno.105276, PMID: 39990210 PMC11840731

[91] Joshi S Sharabi A. Targeting myeloid-derived suppressor cells to enhance natural killer cell-based immunotherapy. Pharmacol Ther. (2022) 235:108114. doi: 10.1016/j.pharmthera.2022.108114, PMID: 35122833 PMC9189042

[92] Chang S Kim YH Kim YJ Kim YW Moon S Lee YY . Taurodeoxycholate increases the number of myeloid-derived suppressor cells that ameliorate Sepsis in mice. Front Immunol. (2018) 9:1984. doi: 10.3389/fimmu.2018.01984, PMID: 30279688 PMC6153344

[93] Sander LE Sackett SD Dierssen U Beraza N Linke RP Müller M . Hepatic acute-phase proteins control innate immune responses during infection by promoting myeloid-derived suppressor cell function. J Exp Med. (2010) 207:1453–64. doi: 10.1084/jem.20091474, PMID: 20530204 PMC2901069

[94] Jia L Long L Wang H Ge C Zhang Z Zhang Z . Early elevation of Monocytic-to-Polymorphonuclear myeloid-derived suppressor cells ratio in critical illness is associated with favorable clinical outcomes. J Inflamm Res. (2025) 18:6807–19. doi: 10.2147/jir.S517333, PMID: 40453971 PMC12126102

[95] He J Zhang Y Jing Y Dong R Li T Zheng X . FXR protects against neonatal sepsis by enhancing the immunosuppressive function of MDSCs. Cell Mol Immunol. (2025) 22:661–73. doi: 10.1038/s41423-025-01289-4, PMID: 40335739 PMC12125261

[96] Cui Z Xu H Wu F Chen J Zhu L Shen Z . Maternal circadian rhythm disruption affects neonatal inflammation via metabolic reprograming of myeloid cells. Nat Metab. (2024) 6:899–913. doi: 10.1038/s42255-024-01021-y, PMID: 38561509

[97] Shi M Chen Z Chen M Liu J Li J Xing Z . Continuous activation of polymorphonuclear myeloid-derived suppressor cells during pregnancy is critical for fetal development. Cell Mol Immunol. (2021) 18:1692–707. doi: 10.1038/s41423-021-00704-w, PMID: 34099889 PMC8245399

[98] Bai AD Lo CKL Komorowski AS Suresh M Guo K Garg A . Staphylococcus aureus bacteraemia mortality: a systematic review and meta-analysis. Clin Microbiol Infect. (2022) 28:1076–84. doi: 10.1016/j.cmi.2022.03.015, PMID: 35339678

[99] Huang X Venet F Wang YL Lepape A Yuan Z Chen Y . PD-1 expression by macrophages plays a pathologic role in altering microbial clearance and the innate inflammatory response to sepsis. Proc Natl Acad Sci USA. (2009) 106:6303–8. doi: 10.1073/pnas.0809422106, PMID: 19332785 PMC2669369

[100] Zhang P Wang Y Yang W Yin Y Li C Ma X . 4-Octyl itaconate regulates immune balance by activating Nrf2 and negatively regulating PD-L1 in a mouse model of sepsis. Int J Biol Sci. (2022) 18:6189–209. doi: 10.7150/ijbs.74456, PMID: 36439878 PMC9682535

[101] Boomer JS To K Chang KC Takasu O Osborne DF Walton AH . Immunosuppression in patients who die of sepsis and multiple organ failure. JAMA. (2011) 306:2594–605. doi: 10.1001/jama.2011.1829, PMID: 22187279 PMC3361243

[102] Yuan Y Hua L Zhou J Liu D Ouyang F Chen X . The effect of artesunate to reverse CLP-induced sepsis immunosuppression mice with secondary infection is tightly related to reducing the apoptosis of T cells via decreasing the inhibiting receptors and activating MAPK/ERK pathway. Int Immunopharmacol. (2023) 124:110917. doi: 10.1016/j.intimp.2023.110917, PMID: 37716165

